# Author Correction: Circular RNA CircFndc3b modulates cardiac repair after myocardial infarction via FUS/VEGF-A axis

**DOI:** 10.1038/s41467-020-15382-x

**Published:** 2020-05-01

**Authors:** Venkata Naga Srikanth Garikipati, Suresh Kumar Verma, Zhongjian Cheng, Dongming Liang, May M. Truongcao, Maria Cimini, Yujia Yue, Grace Huang, Chunlin Wang, Cindy Benedict, Yan Tang, Vandana Mallaredy, Jessica Ibetti, Laurel Grisanti, Sarah M. Schumacher, Erhe Gao, Sudarsan Rajan, Jeremy E. Wilusz, David Goukassian, Steven R. Houser, Walter J. Koch, Raj Kishore

**Affiliations:** 1 Center for Translational Medicine, Lewis Katz School of Medicine, Temple University, Philadelphia, PA 19140 USA; 2 Division of Cardiovascular Diseases, Department of Medicine, The University of Alabama at Birmingham, Birmingham, AL 35294 USA; 3 Department of Biochemistry and Biophysics, University of Pennsylvania Perelman School of Medicine, Philadelphia, PA 19104 USA; 4 Department of Biomedical Sciences, University of Missouri, Columbia, MO 65211 USA; 5 Lerner Research Institute, Cleveland Clinic, Cleveland, Ohio 44195 USA; 6 Zena & Michael A. Weiner Cardiovascular Institute, Icahn School of Medicine at Mount Sinai, New York, NY 10029 USA; 7 Cardiovascular Research Center and Department of Physiology, Lewis Katz School of Medicine, Temple University, Philadelphia, PA 19140 USA; 8 Department of Pharmacology, Lewis Katz School of Medicine, Temple University, Philadelphia, PA 19140 USA

**Keywords:** Non-coding RNAs, Myocardial infarction

Correction to: *Nature Communications* 10.1038/s41467-019-11777-7, published online 20 September 2019.

The original version of this Article contained errors in the references 45, 46, 47, 48, 49 which incorrectly cited:

45. Ji, L. & Roth, J. A. Tumor suppressor FUS1 signaling pathway. *J. Thorac. Oncol.: Off. Publ. Int. Assoc. Study Lung Cancer* **3**, 327–330 (2008).

46. Deng, W. G. et al. Synergistic tumor suppression by coexpression of FUS1 and p53 is associated with down-regulation of murine double minute-2 and activation of the apoptotic protease-activating factor 1-dependent apoptotic pathway in human non-small cell lung cancer cells. *Cancer Res*. **67**, 709–717 (2007).

47. Hesson, L. B., Cooper, W. N. & Latif, F. Evaluation of the 3p21.3 tumour-suppressor gene cluster. *Oncogene* **26**, 7283–7301 (2007).

48. Lin, J. et al. Oncogenic activation of c-Abl in non-small cell lung cancer cells lacking FUS1 expression: inhibition of c-Abl by the tumor suppressor gene product Fus1. *Oncogene* **26**, 6989–6996 (2007).

49. Rimkus, T., Sirkisoon, S., Harrison, A. & Lo, H. W. Tumor suppressor candidate 2 (TUSC2, FUS-1) and human cancers. *Discov. Med.* **23**, 325–330 (2017).

The correct references are:

45. Tan, A.Y., Riley, T.R., Coady, T., Bussemaker, H.J. & Manley, J.L. TLS/FUS (translocated in liposarcoma/fused in sarcoma) regulates target gene transcription via single-stranded DNA response elements. *Proc. Natl Acad. Sci. USA* **109**, 6030–6035 (2012).

46. Ward, C. L. et al. A loss of FUS/TLS function leads to impaired cellular proliferation. *Cell Death Dis.* **5**, e1572 (2014).

47. Crivello, M. et al. Vascular regression precedes motor neuron loss in the FUS (1-359) ALS mouse model. *Dis. Model Mech.* **12**, (2019).

48. Suzuki, H. & Matsuoka, M. Overexpression of nuclear FUS induces neuronal cell death. *Neuroscience* **287**, 113–124 (2015).

49. Brooke, G.N. et al. FUS/TLS is a novel mediator of androgen-dependent cell-cycle progression and prostate cancer growth. *Cancer Res*. **71,** 914–924 (2011).

Consequently, text referring to the incorrect references needed to be corrected. In the sentence “In corroboration with previous reports, we find that circFndc3b interacts with FUS, an RNA binding protein that acts as a tumor suppressor gene in many human cancers45”, the statement “that acts as a tumor suppressor gene in many human cancers45” was incorrect and has been removed.

In the sentence “Furthermore, recent studies demonstrated exogenous FUS gene delivery significantly inhibits tumor growth49 by activating APAF-1 induced apoptosis and inhibiting angiogenesis by reducing VEGF-A expression46”, the statement “by activating APAF-1 induced apoptosis and inhibiting angiogenesis by reducing VEGF-A expression46” was incorrect and has been removed.

This has been corrected in the PDF and HTML versions of the Article.

