# Supplementary material for: Circular RNA CircFndc3b modulates cardiac repair after myocardial infarction via FUS/VEGF-A axis
Source: Nat Commun. 2019 Sep 20;10:4317. doi: 10.1038/s41467-019-11777-7 (PMC6754461; doi:10.1038/s41467-019-11777-7)
Supplement: Supplementary file 1 — Supplementary Information [file 41467_2019_11777_MOESM1_ESM.pdf]

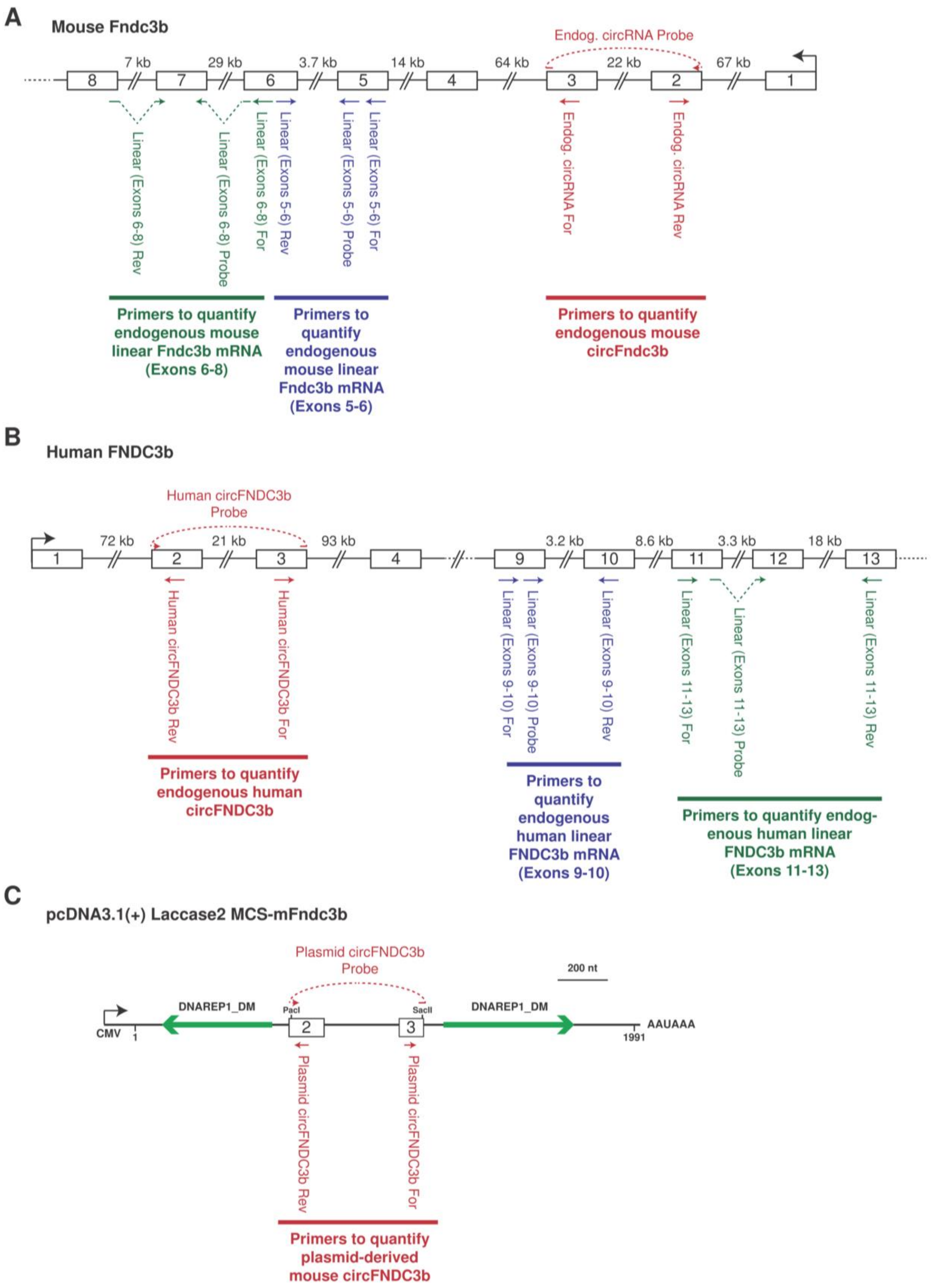
A
Mouse Fndc3b
Endog. circRNA Probe
7 kb
29 kb
3.7 kb
14 kb
64 kb
22 kb
67 kb
8
7
6
5
4
3
2
1
Linear (Exons 6-8) Rev
Linear (Exons 6-8) Probe
Linear (Exons 6-8) For
Linear (Exons 5-6) Rev
Linear (Exons 5-6) Probe
Linear (Exons 5-6) For
Endog. circRNA For
Endog. circRNA Rev
Primers to quantify endogenous mouse linear Fndc3b mRNA (Exons 6-8)
Primers to quantify endogenous mouse linear Fndc3b mRNA (Exons 5-6)
Primers to quantify endogenous mouse circFndc3b
B
Human FNDC3b
Human circFNDC3b Probe
72 kb
21 kb
93 kb
3.2 kb
8.6 kb
3.3 kb
18 kb
1
2
3
4
9
10
11
12
13
Human circFNDC3b Rev
Human circFNDC3b For
Linear (Exons 9-10) For
Linear (Exons 9-10) Probe
Linear (Exons 9-10) Rev
Linear (Exons 11-13) For
Linear (Exons 11-13) Probe
Linear (Exons 11-13) Rev
Primers to quantify endogenous human circFNDC3b
Primers to quantify endogenous human linear FNDC3b mRNA (Exons 9-10)
Primers to quantify endog-enous human linear FNDC3b mRNA (Exons 11-13)
C
pcDNA3.1(+) Laccase2 MCS-mFndc3b
Plasmid circFNDC3b Probe
200 nt
DNAREP1_DM
PacI
SacII
DNAREP1_DM
CMV
1
2
3
1991
AAUAAA
Plasmid circFNDC3b Rev
Plasmid circFNDC3b For
Primers to quantify plasmid-derived mouse circFNDC3b

Supplementary Figure 1: Schematics of Taqman primer sets used to detect and quantify transcripts from **(A)** the endogenous mouse Fndc3b locus, **(B)** the endogenous human FNDC3b locus, and **(C)** the pcDNA3.1(+) Laccase2 MCS-mFndc3b plasmid that is used to overexpress the circular RNA. For the endogenous loci, Taqman primer sets were designed to amplify the circular RNA (red) or the linear mRNA (using exons not within the circular RNA, green and blue).

Supplementary Fig-2

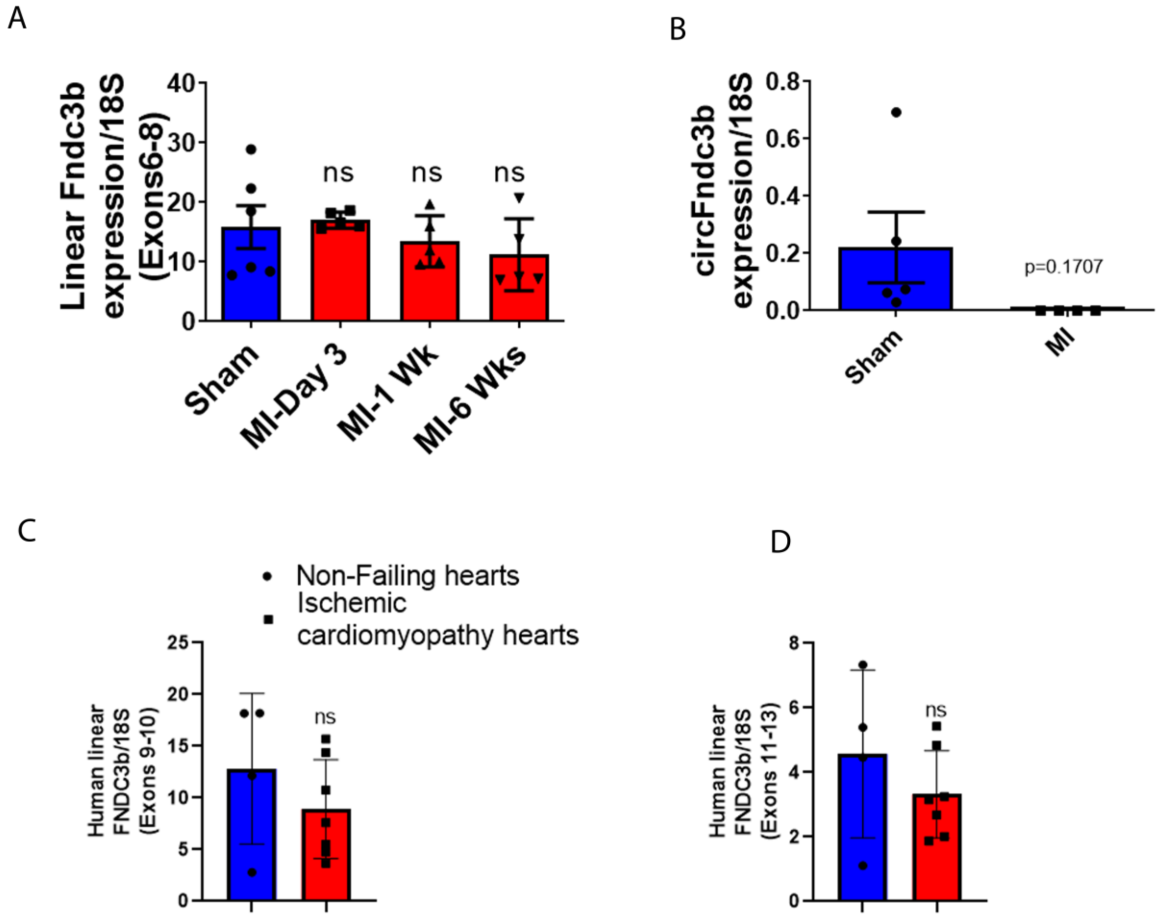

Supplementary Figure 2: CircRNA profiling in sham and MI hearts: **(A)** RT-qPCR analysis of linear Fndc3b expression at different time points in post-MI LV tissues compared to sham, normalized to 18S rRNA. Data are mean ± SEM of n=5-6 animals/group, ns vs sham hearts(one-way ANOVA); **(B)** RT-qPCR analysis of circFndc3b in isolated fibroblasts from 3 days post-MI LV tissue compared to sham controls, normalized to 18S rRNA. Data are mean ± SEM of n = 4-5 group. ns= non-significant vs controls (two-sided unpaired students t-test); **(C-D)** RT-qPCR analysis of human linear Fndc3b in non-failing heart tissues and in heart tissues from patients with ischemic cardiomyopathy, normalized to 18S rRNA. Data are mean ± SEM of n=4-7/group. ns= non-significant vs controls(two-sided unpaired students t-test)

Supplementary Fig.3

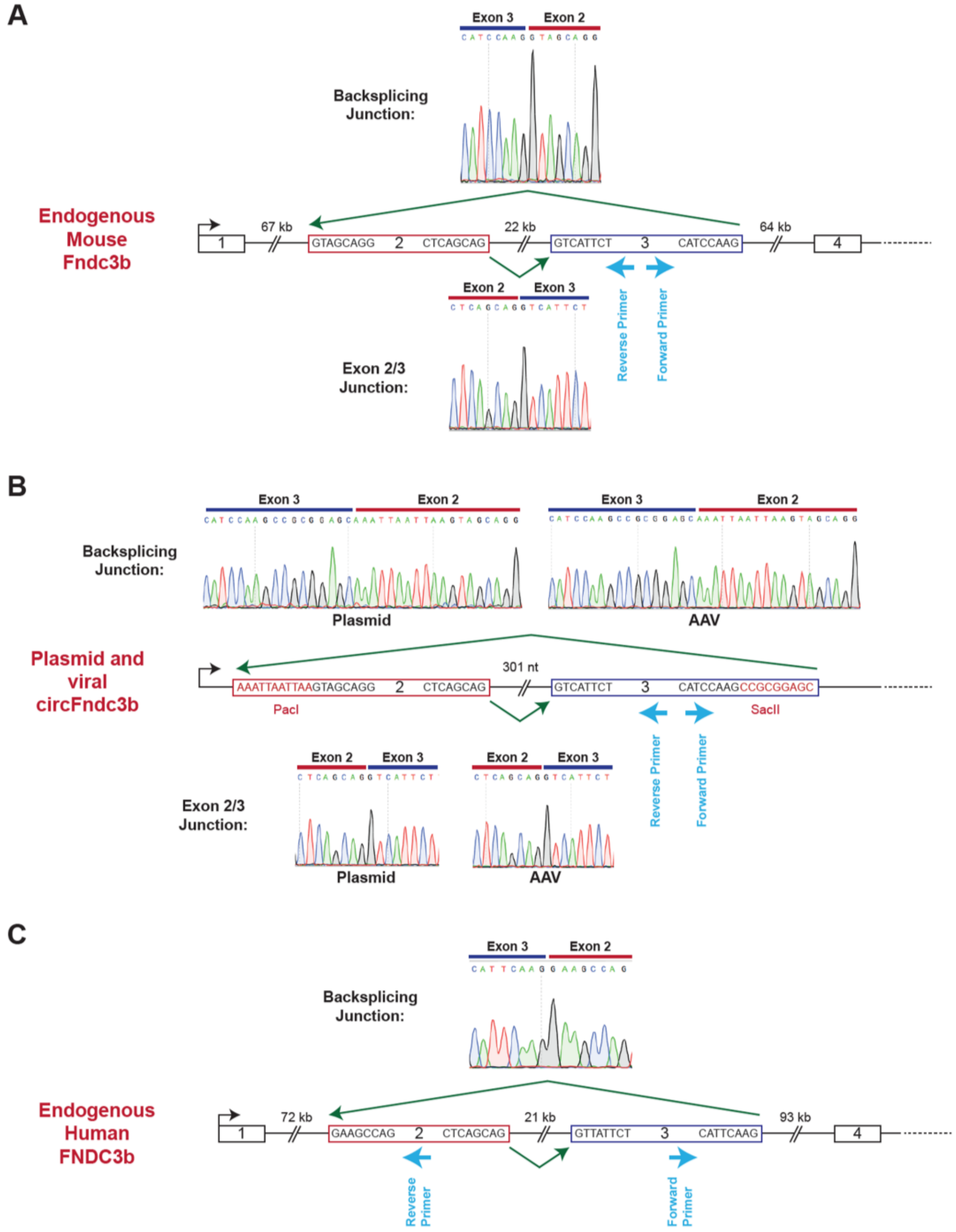

Supplementary Figure 3: Validation of circFndc3b splicing junction by Sanger sequencing: Representative examples of PCR products purified and sequenced to confirm circFndc3b junction sequences of transcripts derived from **(A)** the mouse endogenous Fndc3b gene, **(B)** the circFndc3b overexpression plasmid and AAV9 vector, and **(C)** the endogenous human FNDC3b gene. PCR amplification was performed using the indicated forward and reverse primers, and products were subjected to Sanger sequencing. Exon 2 and 3 sequences are denoted in red and blue, respectively.

Supplementary Fig.4

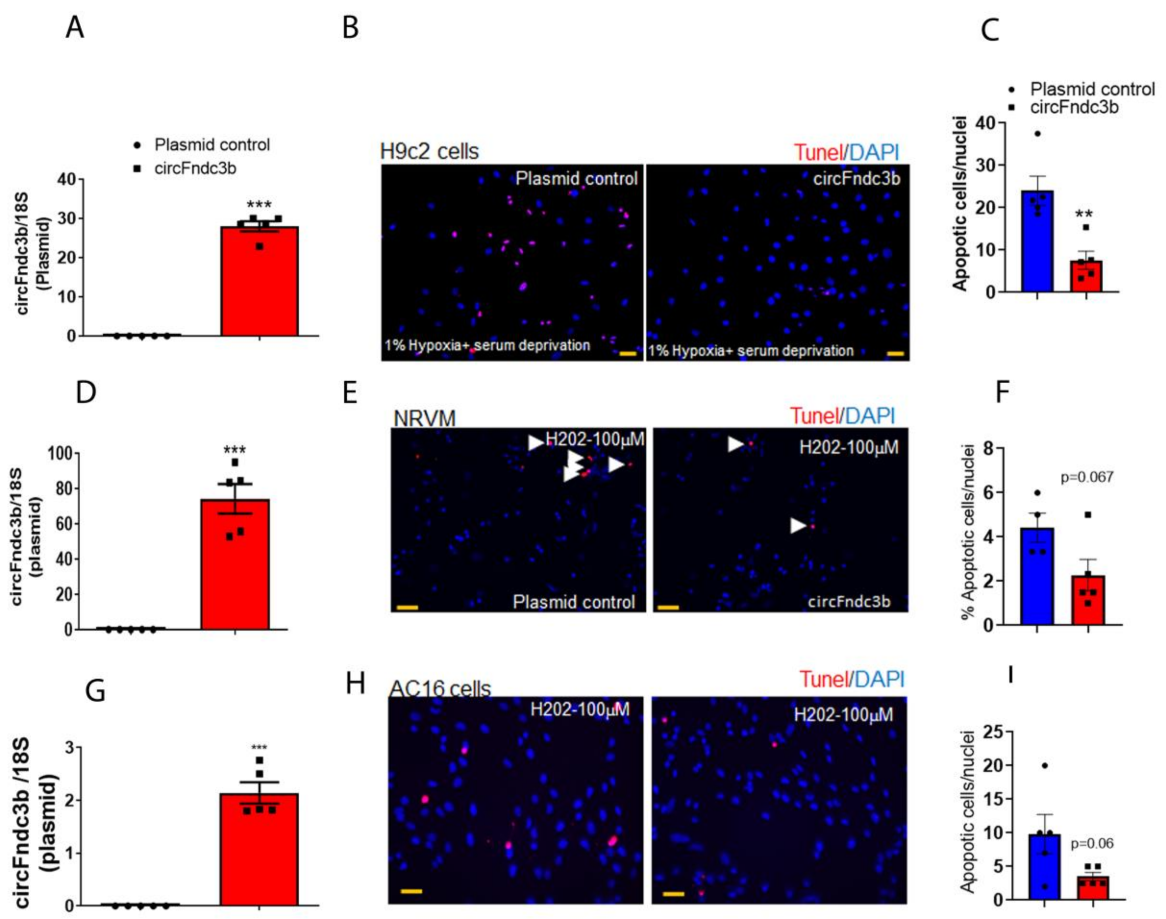

Supplementary Figure 4: circFndc3b reduces apoptosis of cardiomyoblasts/cardiomyocytes *in vitro*: (A) RT-qPCR analysis of circFndc3b in control plasmid or circFndc3b overexpression plasmid treated H9c2, normalized to 18S rRNA. **p<0.01 vs plasmid control; **(B)** Overexpression of circFndc3b reduced hypoxia (1% 0_2, 48h) induced apoptosis (TUNEL+, Red) in H9c2 cells compared to control plasmid treated cells; **(C)** Quantification of TUNEL+ cells presented as the % TUNEL+ positive cells and DAPI-stained nuclei. Data are mean ± SEM of n=5 independent experiments. **p<0.01 vs plasmid control; **(D)** RT-qPCR analysis of circFndc3b in control plasmid or circFNDC3b overexpression plasmid treated NRVM, normalized to 18S rRNA. ***p<0.01 vs plasmid control (two-sided unpaired students t-test); **(E)** Overexpression of circFndc3b reduced H_2O_2 (100μM) induced apoptosis (TUNEL+, Red) in NRVM cells compared to control vector treated cells; **(F)** Quantification of TUNEL+ cells presented as the % TUNEL+ positive cells and DAPI-stained nuclei. Data are mean ± SEM of n=4-5 independent experiments. p=0.067 vs plasmid control; **(G)** RT-qPCR analysis of circFndc3b in control plasmid or circFndc3b overexpression plasmid treated AC16 cells, normalized to 18S rRNA. ***p<0.001 vs plasmid control(two-sided unpaired students t-test); **(H)** Overexpression of circFndc3b reduced H_2O_2 (100μM) induced apoptosis (TUNEL+, Red) in AC16 cells compared to plasmid control treated cells; **(I)** Quantification of TUNEL+ cells presented as

the % TUNEL+ positive cells and DAPI-stained nuclei. Data are mean ± SEM of 5 independent experiments. P=0.06 vs plasmid control (two-sided unpaired students t-test).

Supplementary Fig-5

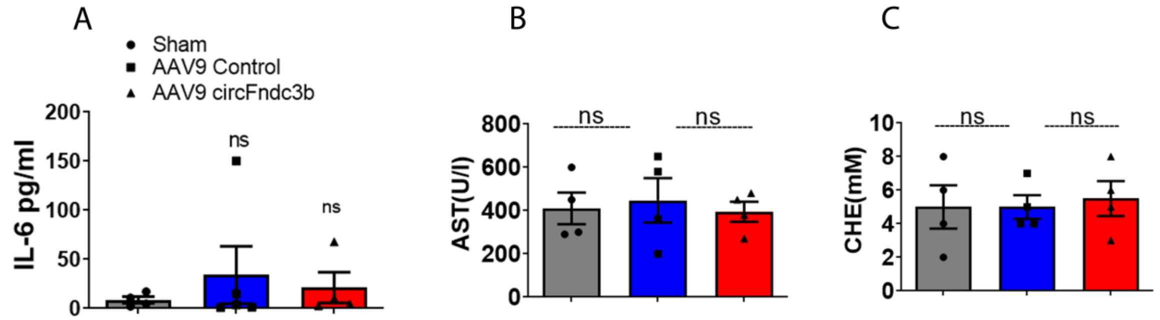

Supplementary Figure 5: CircFndc3b overexpression had no effect on tissue injury markers: (A-C) Bar graphs showing the following clinical chemistry parameters: interleukin 6 (IL-6; a proinflammatory marker), aspartate aminotransferase (AST; liver toxicity marker), and cholinesterase (ChE; neurotoxicity marker) in plasma samples collected 8 weeks post MI in AAV9 control or AAV9 circFndc3b or sham control groups. Data are mean ± SEM of n=4 mice/group. ns= non-significant between the groups(two-sided unpaired students t-test).

Supplementary Fig.6

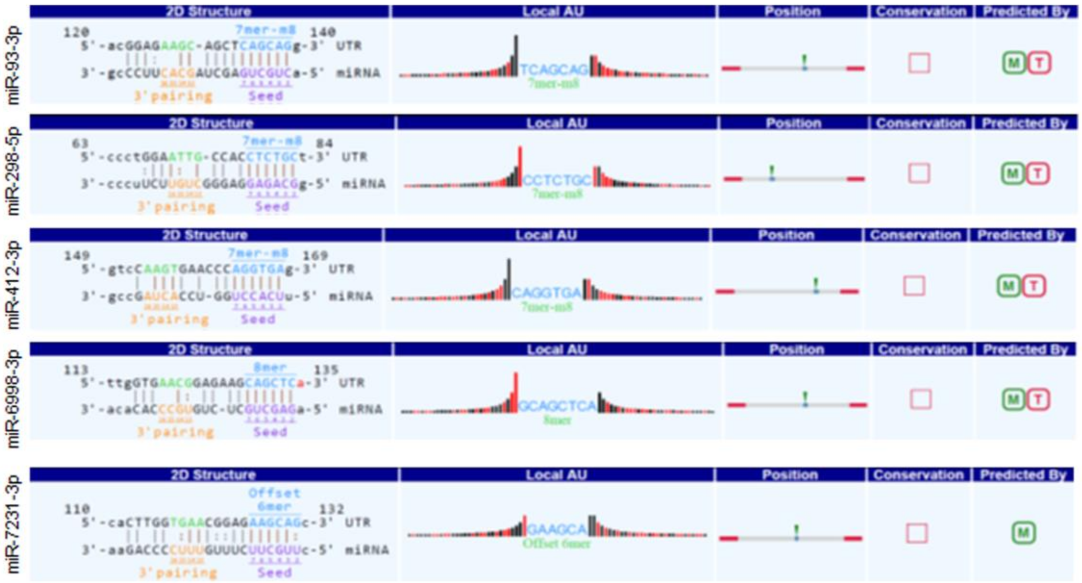

Supplementary Figure 6: CircFndc3b potential miR targets: Arraystar's proprietary program based prediction of circFndc3b target miRNAs.

Supplementary Fig-7

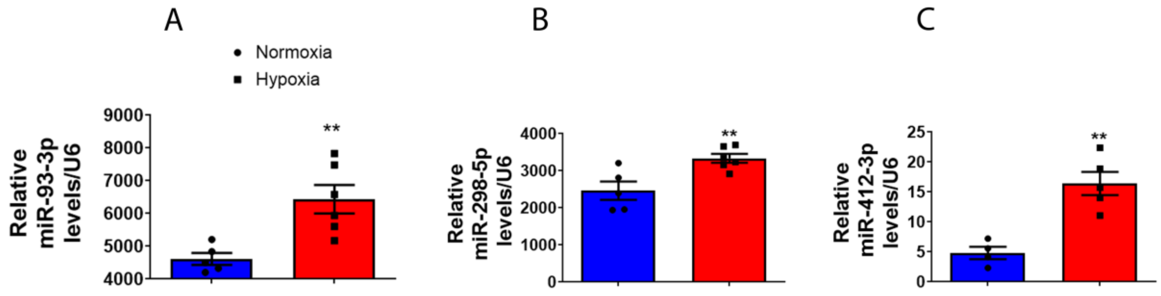

Supplementary Figure 7: miRNA expression under hypoxic conditions: (A-C) RT-qPCR analysis of circFndc3b target miRNAs (miR-93-3p, miR 298-5p, and miR-412-3p) in H9c2 cells subjected to hypoxia stress (1% O_2) for 48h compared to normoxia, data normalized to U6; n=4-6/group. Data are mean ± SEM . **p<0.01 vs normoxia(two-sided unpaired students t-test).

Supplementary Fig-8

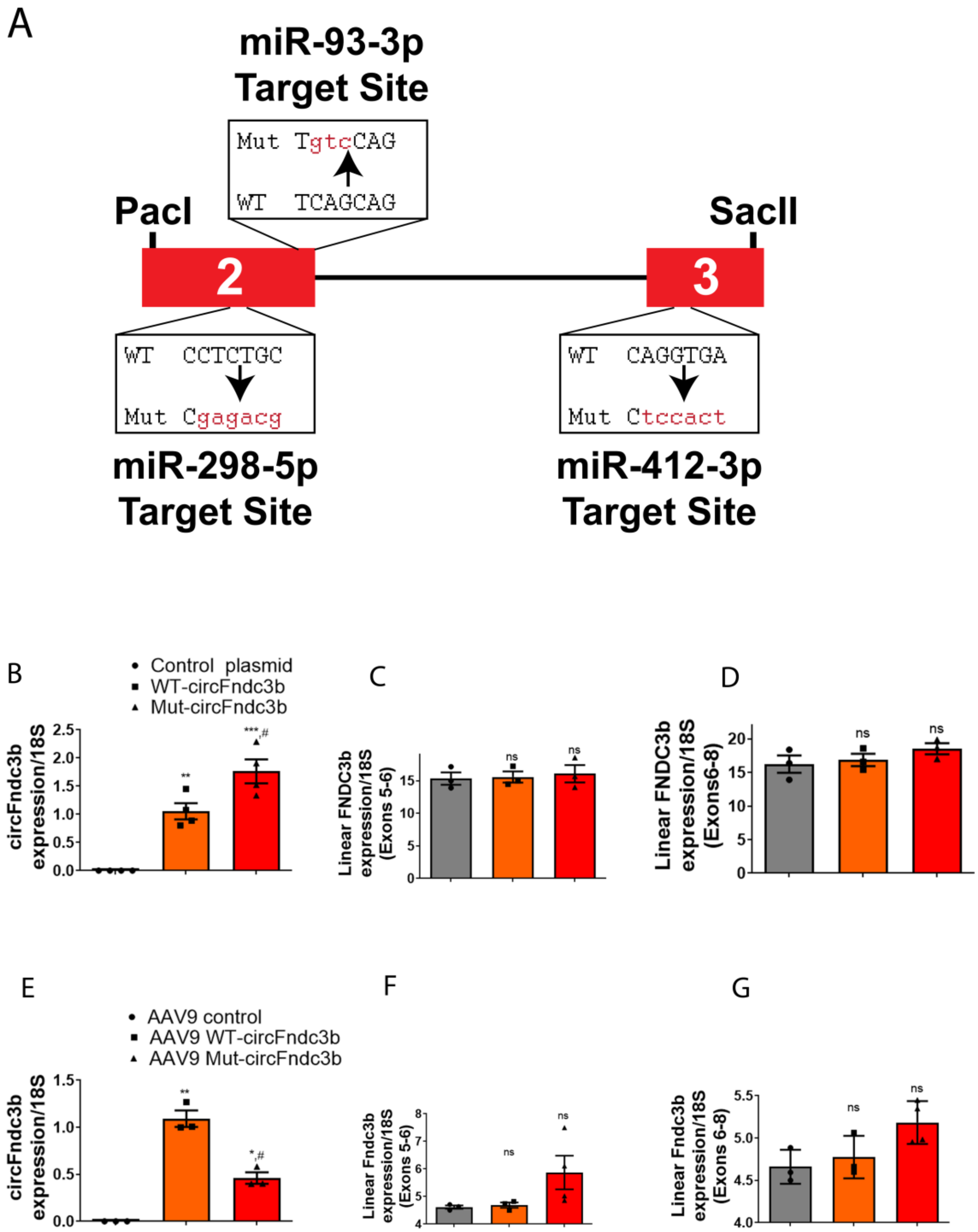

Supplementary Figure 8: Generation of miRNA mutant-circFndc3b overexpression plasmids: (A) Mutated miRNA binding sites in circFndc3b; **(B)** RT-qPCR analysis of WT and Mut-circFndc3b expression in plasmid control or WT-circFndc3b or Mut-circFndc3b overexpression plasmid-treated H9c2 cells, normalized to 18S rRNA. Data are mean ± SEM of n=3-4 independent experiments. $^{**}p<0.01$ $^{***}p<0.001$ vs plasmid control. $\#p<0.05$ vs WT-

circFndc3b treated cells(one way ANOVA); **(C-D)** RT-qPCR analysis of linear Fndc3b mRNA levels in plasmid control or WT or mutant circFndc3b overexpression plasmids-treated cardiac endothelial cells, normalized to 18S rRNA. Data are mean ± SEM of 3 independent experiments. Not significant (ns) vs plasmid control (one way ANOVA; **(E)** RT-qPCR analysis of WT-circFndc3b and Mut-circFndc3b expression in AAV9 control or AAV9 WT-circFndc3b or AAV9 Mut-circFndc3b treated mouse hearts 10 days post-MI, normalized to 18S rRNA. Data are mean ± SEM of 3 independent experiments. *$p<0.05$ **$p<0.01$ vs AAV9 control. #$p<0.05$ vs AAV9 WT-circFndc3b(one way ANOVA); **(F-G)** RT-qPCR analysis of linear Fndc3b mRNA levels in AAV9 control or AAV9 WT-circFndc3b or AAV9 Mut-circFndc3b treated mouse hearts, normalized to 18S rRNA. Data are mean ± SEM of 3 independent experiments. *$p<0.05$ vs plasmid control. Not significant (ns) vs plasmid control (one way ANOVA).

Supplementary Fig.9

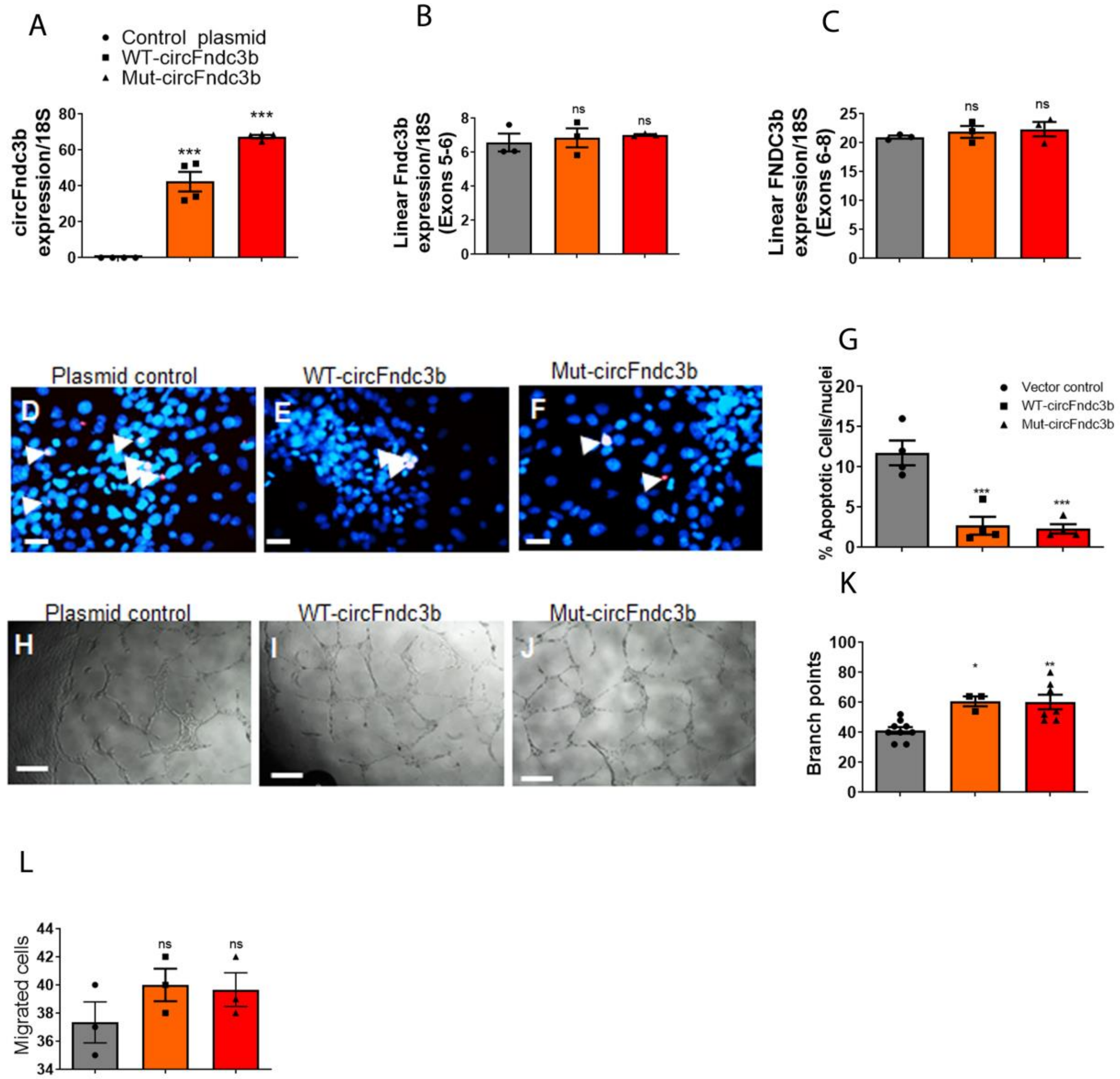

Supplementary Figure 9: CircFndc3b does not act as a miRNA sponge in cardiac endothelial cells *in vitro* (A) RT-qPCR analysis of circFndc3b expression in control plasmid or WT-circFndc3b or Mut-circFndc3b overexpression plasmid-treated endothelial cells, normalized to 18S rRNA. Data are mean ± SEM of n=3-4 independent experiments. ***p<0.001 vs plasmid control (one way ANOVA); **(B-C)** RT-qPCR analysis of linear Fndc3b mRNA expression in control plasmid or WT-circFndc3b or Mut-circFndc3b overexpression plasmid-treated endothelial cells, normalized to 18S rRNA. Data shown are mean ±SEM of three independent experiments. ns-Non-significant vs controls(one way ANOVA); **(D-F)** Overexpression of WT-circFndc3b and Mut-circFndc3b both reduced H_2O_2 (100μM) induced apoptosis (TUNEL+, Red) in mouse cardiac

endothelial cells compared to plasmid control treated cells; **(G)** Quantification of TUNEL+ cells presented as the % TUNEL positive cells per total DAPI-stained nuclei. Data are mean ± SEM of n=3-4 independent experiments. ***p<0.001 vs plasmid control (one way ANOVA); **(H-J)** Matrigel tube formation assay in control or circFndc3b overexpression treated endothelial cells; **(K)** Quantification of branch points. Data are mean ± SEM of n=3-9 independent experiments *p<0.05 **p<0.01 vs plasmid control (one way ANOVA); **(L)** Quantification of MCEC migration in WT-circFndc3b and Mut-circFndc3b treated cells compared to plasmid control treated cells**.** Data are mean ± SEM of 3 independent experiments. ns- Non-significant vs controls (One way ANOVA).

Supplementary Fig-10

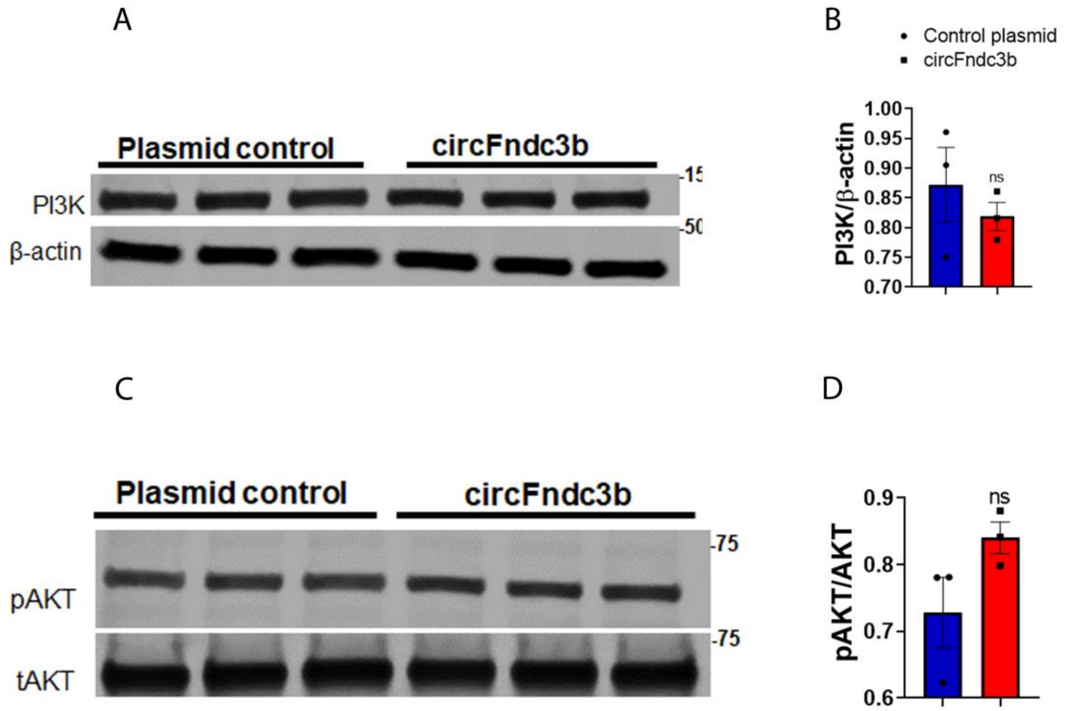

Supplementary Figure 10: circFndc3b does not target the PI3K/AKT signaling axis: **(A, C)** Representative western blot images of PI3K and p-AKT protein levels in MCECs treated with control or circFndc3b overexpression plasmids; **(*B, D*)** Quantification of PI3K and p-AKT levels normalized to β-actin and total AKT respectively. Data are mean ± SEM of 3 independent experiments. ns= non-significant vs control plasmid two-sided unpaired students t-test.

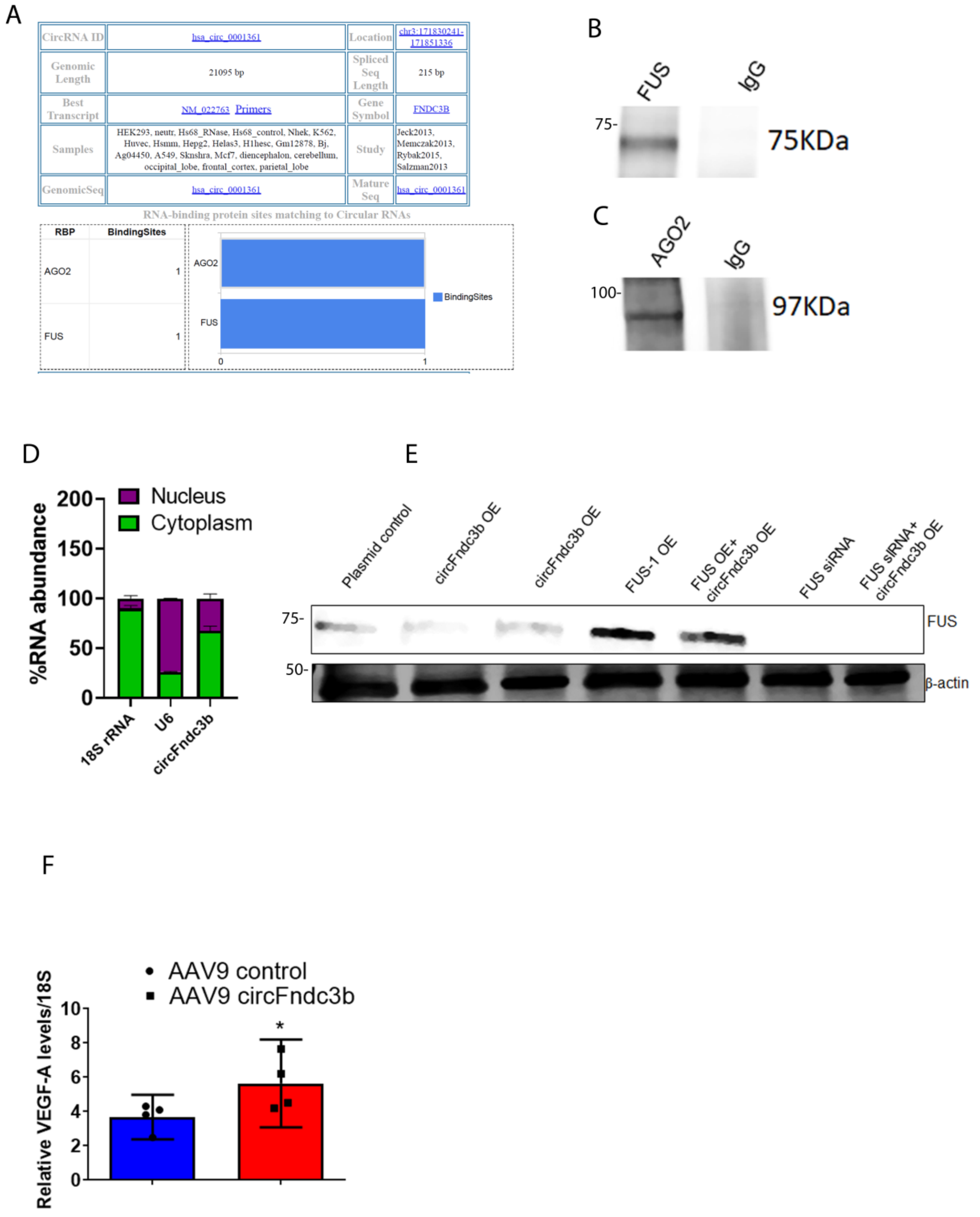
A
CircRNA ID
hsa_circ_0001361
Location
chr3:171830241-171851336
Genomic Length
21095 bp
Spliced Seq Length
215 bp
Best Transcript
NM_022763 Primers
Gene Symbol
FNDC3B
Samples
HEK293, neutr, Hs68_RNase, Hs68_control, Nhek, K562, Huvec, Hsmm, Hepg2, Helas3, H1hesc, Gm12878, Bj, Ag04450, A549, Sknshra, Mcf7, diencephalon, cerebellum, occipital_lobe, frontal_cortex, parietal_lobe
Study
Jeck2013, Memczak2013, Rybak2015, Salzman2013
GenomicSeq
hsa_circ_0001361
Mature Seq
hsa_circ_0001361
RNA-binding protein sites matching to Circular RNAs
RBP
BindingSites
AGO2
1
FUS
1
BindingSites
B
FUS
IgG
75-
75KDa
C
AGO2
IgG
100-
97KDa
D
Nucleus
Cytoplasm
%RNA abundance
200
150
100
50
0
18S rRNA
U6
circFndc3b
E
Plasmid control
circFndc3b OE
circFndc3b OE
FUS-1 OE
FUS OE+ circFndc3b OE
FUS siRNA
FUS siRNA+ circFndc3b OE
75-
FUS
50-
β-actin
F
AAV9 control
AAV9 circFndc3b
Relative VEGF-A levels/18S
10
8
6
4
2
0
*

Supplementary Figure 11: circFndc3b-FUS-VEGF-A signaling network regulates cardiac endothelial cell function: (A) *In silico* prediction of circFndc3b binding to FUS and AGO2 using circinteractome program; **(B-C)** Representative western blot images showing immunoprecipitation of FUS and AGO2 in MCECs and IgG as control; **(D)** RT-PCR quantification of circFndc3b in RNA from MCEC nuclear and cytoplasmic fractions. Cytoplasmic fraction was normalized to 18S rRNA and nuclear fraction was normalized to U6 snRNA. Data are mean ± SEM of 3 independent experiments; **(E)** MCECs were transfected alone or in combination FUS siRNA or FUS overexpression plasmid or circFNDC3b overexpression plasmid or their respective controls for 48 h; **(E)** Representative western blot images of FUS normalized to β-actin. Data are mean ± SEM of 3 independent experiments; **(F)** RT-qPCR analysis of VEGF-A in AAV9 control or AAV9 circFNDC3b treated mouse hearts 8 weeks post-MI, normalized to 18S. Data are mean ± SEM of n=4/group. *p<0.05 vs AAV9 control two-sided unpaired students t-test.

Supplementary Table-1: Demographic details of the human LV tissue samples

Age (Ranging)	Sex	Etiology
27-73	3-Female<br>1-Male	Non-failing
27-68	7-Male	Ischemic

Supplementary Table-2: Primers used in this study:

Mouse circFndc3b (endogenous)	Forward	CCTTTACCATCAGAGCCGAG
	Reverse	CATCATGGTGACGTACATTCATG
	Probe	/56-FAM/TGCATCCA/ZEN/AGGTAGCAGGTTGAG/3IABkFQ/
Mouse circFndc3b (plasmid)	Forward	CCTTTACCATCAGAGCCGAG
	Reverse	CATCATGGTGACGTACATTCATG
	Probe	/56-FAM/CATCCAAGC/ZEN/CGCGGAGCAAATTAA/3IABkFQ/
Mouse Linear Fndc3b (Exons 5-6)	Forward	GAGATATGCCACCCCAGTT
	Reverse	TAGCTCGACATTCCATACAGG
	Probe	/56-FAM/CAGCCTCAC/ZEN/CTTCCTCCCACAATA/3IABkFQ/
Mouse Linear Fndc3b (Exons 6-8)	Forward	ATCAAGAAGACAGAGCGAAGAG
	Reverse	CCTGGATATTAGACACCTGTGG
	Probe	/56-FAM/CTGACCTGC/ZEN/AAGAGTATGAGTTGGAATT/3IABkFQ/
Human circFNDC3b	Forward	TCACAATAAGAGCAGAGGATGG
	Reverse	GGCAGTTCCAGAGGGATTT
	Probe	/56-FAM/AGTGCATTC/ZEN/AAGGAAGCCAGTTGAG/3IABkFQ/
	Forward	GAAGCACTGTCTGAAACCACTA

Human Linear FNDC3b (Exons 11-13)	Reverse	GGCACATAGGAGCAAAAGTTC
	Probe	/56-FAM/CTGCAGTGG/ZEN/AAGGCACCAATTGAC/3IABkFQ/
Human Linear FNDC3b (Exons 9-10)	Forward	GCTGGTCTAAGATCTTTCAGGT
	Reverse	GTCTTTCCTTCCCCTACAGTT
	Probe	/56-FAM/AGGTGGCCT/ZEN/TATCAGACAAAGGACG/3IABkFQ/
circJmjd1c	Forward	ATTCCTTATAGATAAGCAACTGGAT
	Reverse	CCCGTTTATCCCACTCAAG
	Probe	56-FAM/CCATACCAG/ZEN/GTCTACGTGGAATTTG/3IABkFQ/
circMcu	Forward	GTATTGGTGATGGGACCTGTT
(SYBR Green chemistry)	Reverse	TGCCAAGTAAGCAAAGACCA
circSenp6	Forward	ACGCCAGGCATCAGAAC
	Reverse	TTTAAAGCCTCCATCCCTCT
	Probe	56-FAM/CGGCTCAGA/ZEN/GCTTTGGATAGATCAG/3IABkFQ/

circHnrnpu	Forward	CCAACAGAGGGAACTATAACCA
	Reverse	GCATTCCACCTCTGTTGTAATT
	Probe	/56-FAM/AGAGGACGA/ZEN/GGAAATAATCGTGGTTAC/3IABkFQ/
Vegfa	Forward	AGGCTGCTGTAACGATGAAG
	Reverse	TCTCCTATGTGCTGGCTTTG
	Probe	/56-FAM/CCACGTCAG/ZEN/AGAGCAACATCACCA/3IABkFQ/
FUS	Forward	GAAGCAGTGGTGGTGGCTATGAA
	Reverse	CTTCAGGAGCCAGGCTAATATG
18S rRNA	Forward	ACGAGATCTGGCATGCTAACTAGT
	Reverse	CGCCACTTGTCCCTCTAAGAA
	Probe	/56-FAM/ACGCGACCC/ZEN/CCGAGCGGT/3IABkFQ/
miR-93-3p	Assay ID: 002231	Thermofisher
miR-298-5P	Assay ID: 002598	Thermofisher
miR-412-3p	Assay ID: 002575<br><br>Assay ID: 463026_mat	Thermofisher
U6 snRNA	Assay ID:	Thermofisher

	001973	
**Primers for validating splice junctions**		
Mouse circFndc3b (endogenous)	Forward	GCCGAGGATGGGACACTTC
	Reverse	CTCACCTGGGTTCACTTGGA
Mouse circFndc3b (Plasmid)	Forward	TTCAGTGCATCCAAGCCGC
	Reverse	CTGGGTTCACTTGGACAAGAA
Human circFNDC3b (endogenous)	Forward	TCACAATAAGAGCAGAGGATGG
	Reverse	GGCAGTTCCAGAGGGATTT

Supplementary Table 3: Echocardiography Measurements

	MI+Saline					MI+ AAV9 control					MI+AAV9 circFNDC3b				
	Baseline (n=5)	1Wk (n=5)	2Wks (n=5)	4Wks (n=5)	8Wks (n=5)	Baseline (n=6)	1Wk (n=6)	2Wks (n=6)	4Wks (n=6)	8Wks (n=6)	Baseline (n=7)	1Wk (n=7	2Wks (n=7)	4Wks (n=7)	8Wks (n=7)
**LVID;d**	3.06±0.29	4.05±0.59	4.42±0.05	5.57±0.20	4.46±0.23	3.23±0.50	5.17±0.50	5±0.98	5.53±1.10	5.05±0.69	3.26±0.31	4.15±1.72	4.62±0.54	4.11±0.52	4.68±0.43
**LVPW;d**	1.468±0.19	1.06±0.46	0.94±8.05	0.80±0.20	1.5±0.13	1.09±0.17	0.84±0.36	1.04±0.35	1.13±0.41	0.81±0.31	1.21±0.30	0.80±0.45	1.51±0.39	1.59±0.32	1.59±0.24
**LVID;s**	2.07±0.24	3.52±0.56	4.04±7.05	4.93±0.17	4.07±0.28	2.167±0.39	4.71±0.41	4.53±0.90	5.03±1.14	4.50±0.62	2.16±0.22	3.77±1.51	4.00±0.46	3.31±0.48	3.76±0.42
**LVPW;s**	1.618±0.17	1.01±0.54	0.97±7.05	0.83±0.29	1.59±0.18	1.44±0.22	1.13±0.46	1.16±0.36	1.19±0.31	0.92±0.42	1.62±0.24	0.91±0.41	1.52±0.29	1.91±0.32	2.04±0.32
**LVAW;d**	0.836±0.30	1.04±0.14	0.89±8.05	0.83±0.34	0.92±0.11	0.80±0.32	0.83±0.37	0.87±0.35	0.76±0.34	0.75±0.26	0.92±0.33	0.89±0.37	1.22±0.27	1.26±0.20	1.24±0.28
**LVAW;s**	1.284±0.2	1.25±0.18	1.01±0.055	1.3±0.15	1.07±0.25	1.20±0.36	0.95±0.46	1.07±0.46	0.93±0.46	0.89±0.31	1.36±0.24	1.10±0.49	1.54±0.30	1.58±0.22	1.53±0.16
**LV Vol;d**	37.26±8.2	73.94±25.13	90.31±9.05	152.35±12.9	91.25±11.68	43.29±15.22	129.24±26.67	123.71±51.30	156.26±64.53	123.42±39.84	43.39±10.18	102.11±34.93	100.39±26.62	76.09±22.55	102.47±22.49
**LV Vol;s**	14.24±4.12	53.22±19.60	73.01±5.05	114.82±9.88	73.35±11.74	16.49±7.03	103.59±19.45	98.25±42.62	126.96±61.71	94.36±30.90	15.68±4.34	80.28±30.26	71.24±19.10	45.72±15.81	61.45±16.71
**%EF**	61.79±6.2	28.75±5.51	18.83±6.0	24.58±2.8	19.64±8.506	62.8±3.95	19.38±4.72	20.86±4.71	20.69±7.74	23.40±6.48	62.51±8.91	22.33±3.10	28.93±3.97	40.61±5.09	40.20±8.31
**% FS**	32.33±4.7	13.24±2.65	8.47±9.05	11.49±1.4	8.938±4.02	33.06±2.52	8.88±2.32	9.54±2.29	9.56±3.70	10.86±3.21	33.19±6.97	10.23±1.44	13.50±2.07	19.59±2.74	19.68±4.59
**LV Mass (AW)**	136.91±48.6	172.59±16.7	172.27±5.0	219.99±96	258.82±16.13	113.82±48.80	196.64±100.17	206.69±72.34	248.91±46.46	168.95±59.92	130.64±45.14	210.11±76.03	323.52±91.73	282.25±57.72	337.08±50.53
**LV Mass (AW) Corrected**	109.53±38.9	138.07±13.37	137.81±0.05	175.9±76.8	207.05±12.90	91.06±39.04	157.31±80.13	165.35±57.87	199.13±37.17	135.16±47.94	104.51±36.11	168.09±60.83	258.82±73.39	225.79±46.18	269.67±40.42
