# Supplementary material for: Circular RNA CircFndc3b modulates cardiac repair after myocardial infarction via FUS/VEGF-A axis
Source: Nat Commun. 2019 Sep 20;10:4317. doi: 10.1038/s41467-019-11777-7 (PMC6754461; doi:10.1038/s41467-019-11777-7)
Supplement: Supplementary file 3 — Reporting Summary [file 41467_2019_11777_MOESM3_ESM.pdf]

nature research

Corresponding author(s): *Double-blind peer review submissions: write DBPR and your manuscript number here instead of author names.*

Last updated by author(s): *YYYY-MM-DD*

Reporting Summary

Nature Research wishes to improve the reproducibility of the work that we publish. This form provides structure for consistency and transparency in reporting. For further information on Nature Research policies, see Authors & Referees and the Editorial Policy Checklist.

Statistics

For all statistical analyses, confirm that the following items are present in the figure legend, table legend, main text, or Methods section.

n/a	Confirmed	
☐	☒	The exact sample size (n) for each experimental group/condition, given as a discrete number and unit of measurement
☐	☒	A statement on whether measurements were taken from distinct samples or whether the same sample was measured repeatedly
☐	☒	The statistical test(s) used AND whether they are one- or two-sided<br>*Only common tests should be described solely by name; describe more complex techniques in the Methods section.*
☐	☒	A description of all covariates tested
☐	☒	A description of any assumptions or corrections, such as tests of normality and adjustment for multiple comparisons
☐	☒	A full description of the statistical parameters including central tendency (e.g. means) or other basic estimates (e.g. regression coefficient) AND variation (e.g. standard deviation) or associated estimates of uncertainty (e.g. confidence intervals)
☒	☐	For null hypothesis testing, the test statistic (e.g. F, t, r) with confidence intervals, effect sizes, degrees of freedom and P value noted<br>*Give P values as exact values whenever suitable.*
☒	☐	For Bayesian analysis, information on the choice of priors and Markov chain Monte Carlo settings
☒	☐	For hierarchical and complex designs, identification of the appropriate level for tests and full reporting of outcomes
☒	☐	Estimates of effect sizes (e.g. Cohen's d, Pearson's r), indicating how they were calculated

Our web collection on statistics for biologists contains articles on many of the points above.

Software and code

Policy information about availability of computer code

Data collection	A detailed description of the software has been included in the Methods section
Data analysis	A detailed description of the software has been included in the Methods section

For manuscripts utilizing custom algorithms or software that are central to the research but not yet described in published literature, software must be made available to editors/reviewers. We strongly encourage code deposition in a community repository (e.g. GitHub). See the Nature Research guidelines for submitting code & software for further information.

Data

Policy information about availability of data

All manuscripts must include a data availability statement. This statement should provide the following information, where applicable:

- Accession codes, unique identifiers, or web links for publicly available datasets
- A list of figures that have associated raw data
- A description of any restrictions on data availability

The microarray data that support the findings of this study are available at Gene Expression Omnibus (GEO), with the accession number GSE133503. The source data underlying Figs.1B-E, 1G, 2B-E, 3B, 3D, 3G, 4B-I, 5B-C, 6A-C, 6H, 6J, 7B-L. Supplementary figs 2A-D, 4A, 4C, 4D, 4F-I, 5A-C, 7A-C, 8C-G,9A-C, 9G, 9K-L, 10A-D, 11B-11D, 11F are provided as source data file.

Field-specific reporting

Please select the one below that is the best fit for your research. If you are not sure, read the appropriate sections before making your selection.

☒ Life sciences ☐ Behavioural & social sciences ☐ Ecological, evolutionary & environmental sciences

For a reference copy of the document with all sections, see nature.com/documents/nr-reporting-summary-flat.pdf

Life sciences study design

All studies must disclose on these points even when the disclosure is negative.

Sample size	No sample size estimation was performed for experiments involving primary cells and mouse experiments . Sample size was based on the magnitude of effect observed, whether or not statistical significance was reached
Data exclusions	No data exclusions
Replication	Data were representative of three or more experiments. The number of replicates is presented in each figure legend
Randomization	C57BL/6 mice were randomly allocated to groups before different treatments
Blinding	In vivo experiments were blinded for the surgeon performing MI or sham surgeries. Similarly for echocardiography.

Reporting for specific materials, systems and methods

We require information from authors about some types of materials, experimental systems and methods used in many studies. Here, indicate whether each material, system or method listed is relevant to your study. If you are not sure if a list item applies to your research, read the appropriate section before selecting a response.

Materials & experimental systems

n/a	Involved in the study
☐	☒ Antibodies
☐	☒ Eukaryotic cell lines
☒	☐ Palaeontology
☐	☒ Animals and other organisms
☐	☒ Human research participants
☒	☐ Clinical data

Methods

n/a	Involved in the study
☒	☐ ChIP-seq
☒	☐ Flow cytometry
☒	☐ MRI-based neuroimaging

Antibodies

Antibodies used	βactin Cell Signaling Technologies 3700<br>FUS -Cell Signaling technologies# 4885s<br>VEGF -Abcam #181300<br>Argonoute-2- Cell Signaling Technologies# 2897<br>PI 3-kinase p110δ Antibody- Santa Cruz: sc-55589<br>Phospho-Akt (Thr308) Cell Signaling Technologies# #9275<br>Akt Antibody- Cell signaling technologies #9272<br>CD31 R&D systems AF3628<br>α-sarcomeric<br>Actin Sigma Aldrich A2172<br>α-smooth muscle Actin Sigma Aldrich A2547<br>Donkey anti-Goat IgG (H+L) Cross-Adsorbed Secondary Antibody, Alexa Fluor 555 Thermofisher A-21432<br>Donkey anti-Mouse IgG (H+L) Highly Cross-Adsorbed Secondary Antibody, Alexa Fluor 488 Thermofisher A-21202
Validation	Antibodies used in this study are commercially available,the specificity had been tested by the supplier

Eukaryotic cell lines

Policy information about cell lines

Cell line source(s)	AC16 cells Millipore-Sigma SCC109<br>Mouse cardiac endothelial cell line CEDARLANE CLU510

	Human vascular endothelial cells ATCC CRL1730<br>H9c2 cells ATCC CRL-1446<br>Mouse primary cells cardiomyocytes, endothelial cells and fibroblasts were isolated as described in the methods
Authentication	Cell lines used in this study are commercially available,the specificity had been tested by the supplier
Mycoplasma contamination	All cell lines were tested and were found negative.
Commonly misidentified lines<br>(See ICLAC register)	No commonly misidentified cell lines were used.

Animals and other organisms

Policy information about studies involving animals; ARRIVE guidelines recommended for reporting animal research

Laboratory animals	Mouse lines were all in the C57BL6/J background.
Wild animals	N/A
Field-collected samples	N/A
Ethics oversight	This study conforms to the Guide for the Care and Use of Laboratory Animals published by the US National Institutes of Health. All experiments conform to the protocols approved by the Institutional Animal Care and Use Committee of Temple University School of Medicine

Note that full information on the approval of the study protocol must also be provided in the manuscript.

Human research participants

Policy information about studies involving human research participants

Population characteristics	Heart tissue samples (n=7) were obtained from ischemic cardiomyopathy patients at the time of transplantation at the Temple University Cardiovascular Research Center, Philadelphia, Pennsylvania and immediately frozen in liquid nitrogen and stored at -80°C until use. Non-failing heart (n=4) tissues were obtained from donor hearts not used for transplantation and were collected and stored in the same manner.
Recruitment	Subjects remain unidentified. Heart samples were collected from tissue bio-bank.
Ethics oversight	The study was conducted in accordance with the Declaration of Helsinki. All tissues were collected with patients' consent for research purposes and the protocol was approved by the Temple University Institutional Review Board.

Note that full information on the approval of the study protocol must also be provided in the manuscript.
