# Supplementary figures and images for: Circular RNA CircFndc3b modulates cardiac repair after myocardial infarction via FUS/VEGF-A axis
Source: Nat Commun. 2019 Sep 20;10:4317. doi: 10.1038/s41467-019-11777-7 (PMC6754461; doi:10.1038/s41467-019-11777-7)

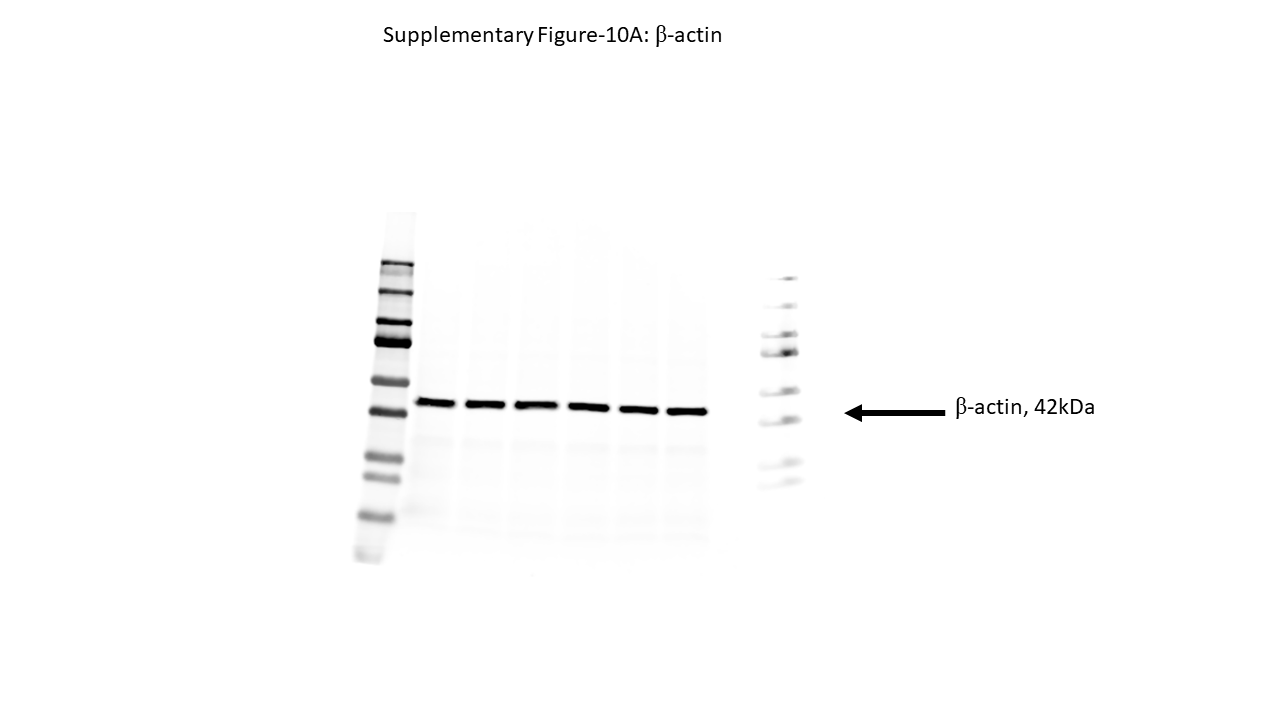

Supplement: Supplementary file 4 — Source Data [file 41467_2019_11777_MOESM4_ESM.zip › Source data/Slide9.TIF]

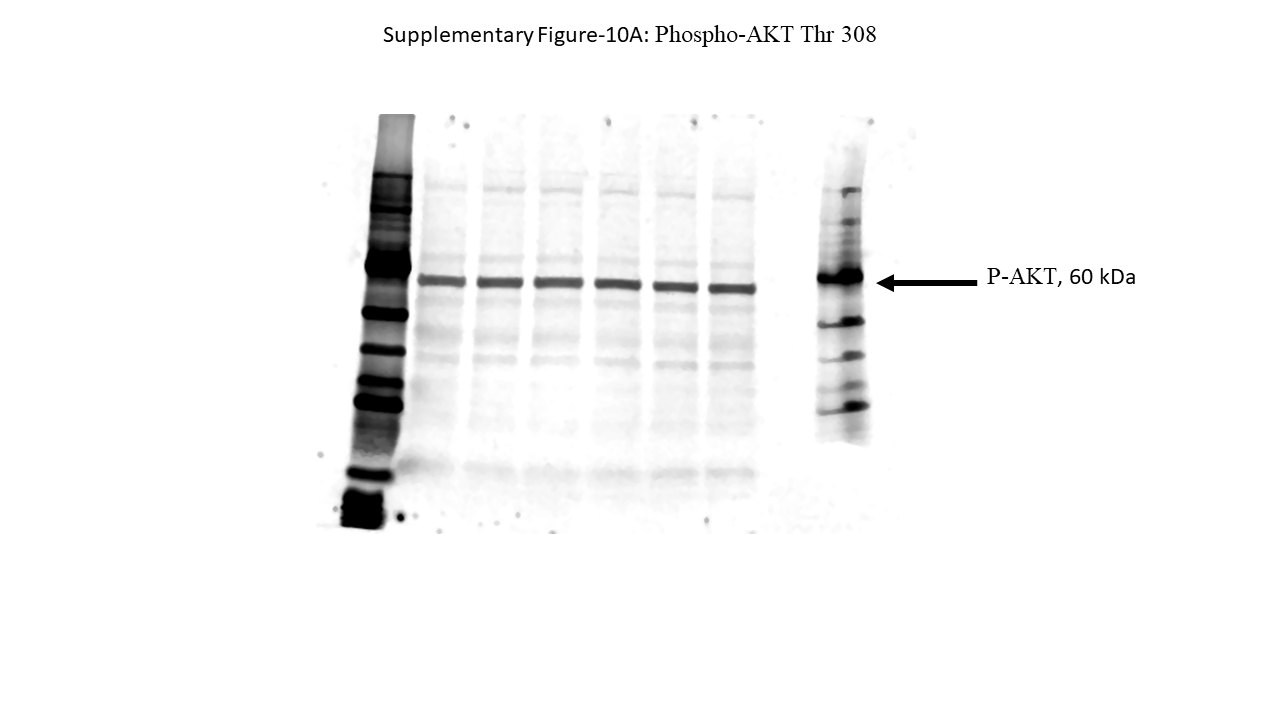

Supplement: Supplementary file 4 — Source Data [file 41467_2019_11777_MOESM4_ESM.zip › Source data/Slide10.TIF]

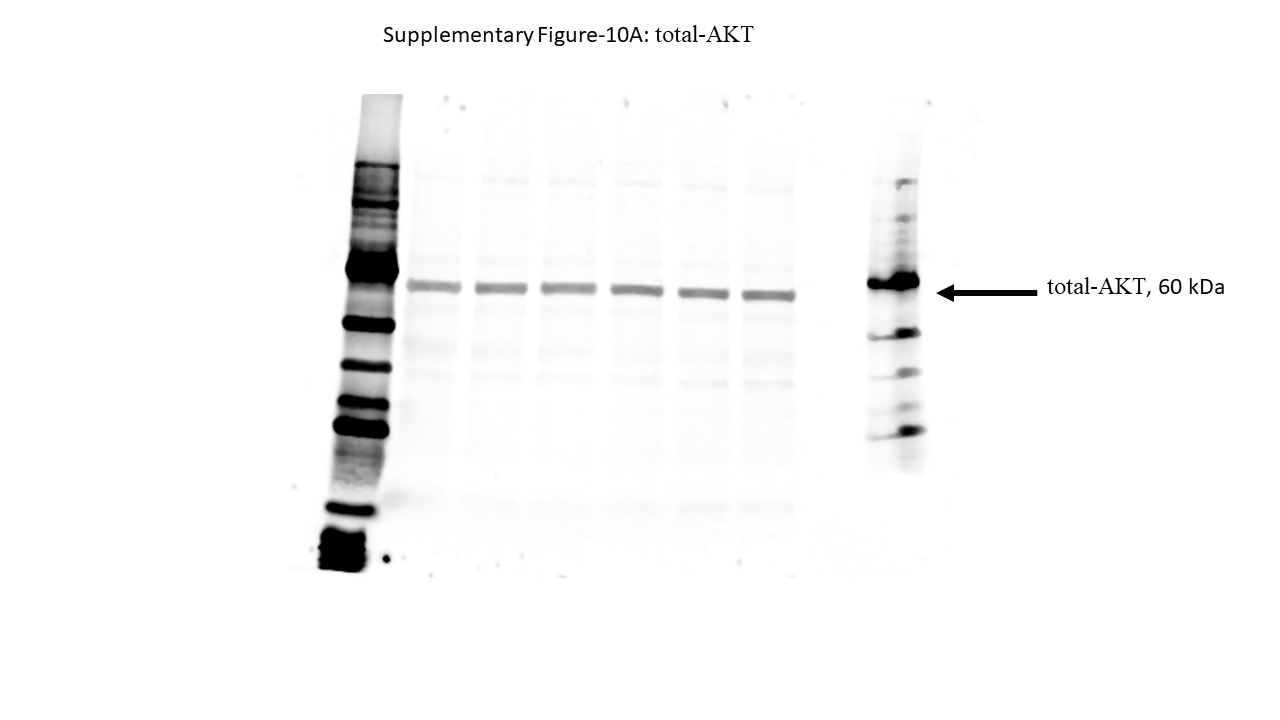

Supplement: Supplementary file 4 — Source Data [file 41467_2019_11777_MOESM4_ESM.zip › Source data/Slide11.TIF]

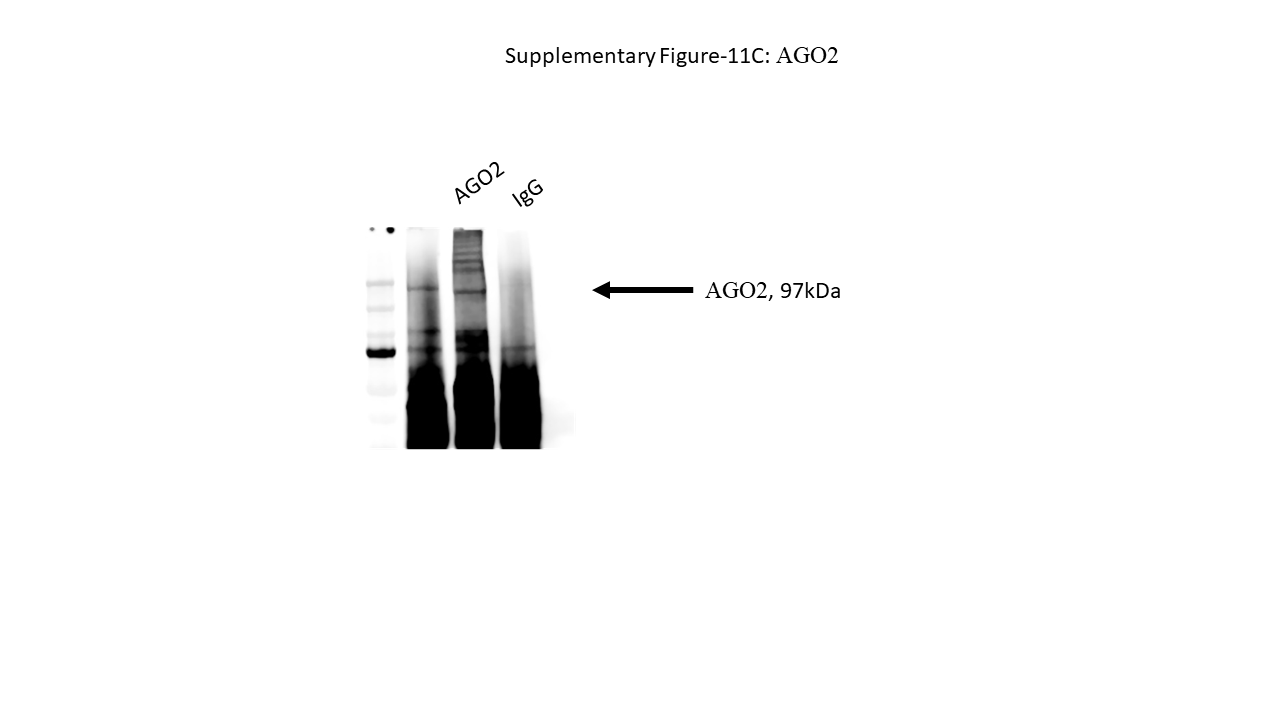

Supplement: Supplementary file 4 — Source Data [file 41467_2019_11777_MOESM4_ESM.zip › Source data/Slide13.TIF]

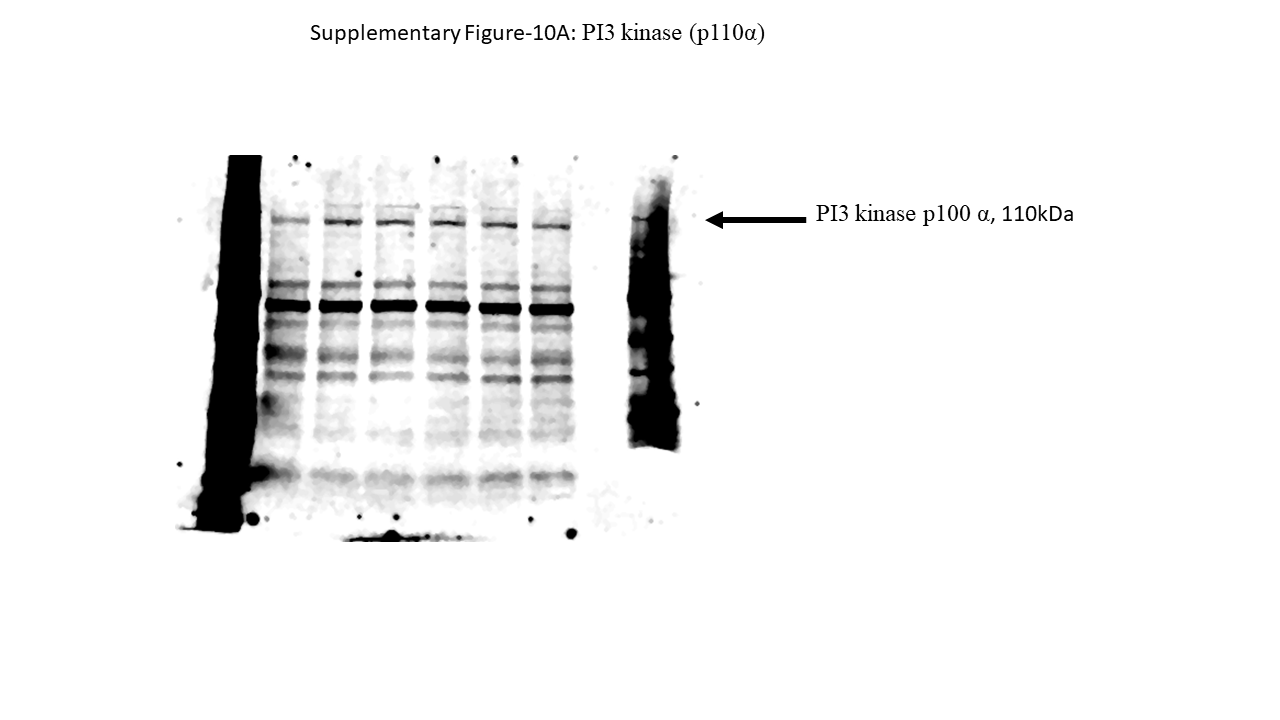

Supplement: Supplementary file 4 — Source Data [file 41467_2019_11777_MOESM4_ESM.zip › Source data/Slide8.TIF]

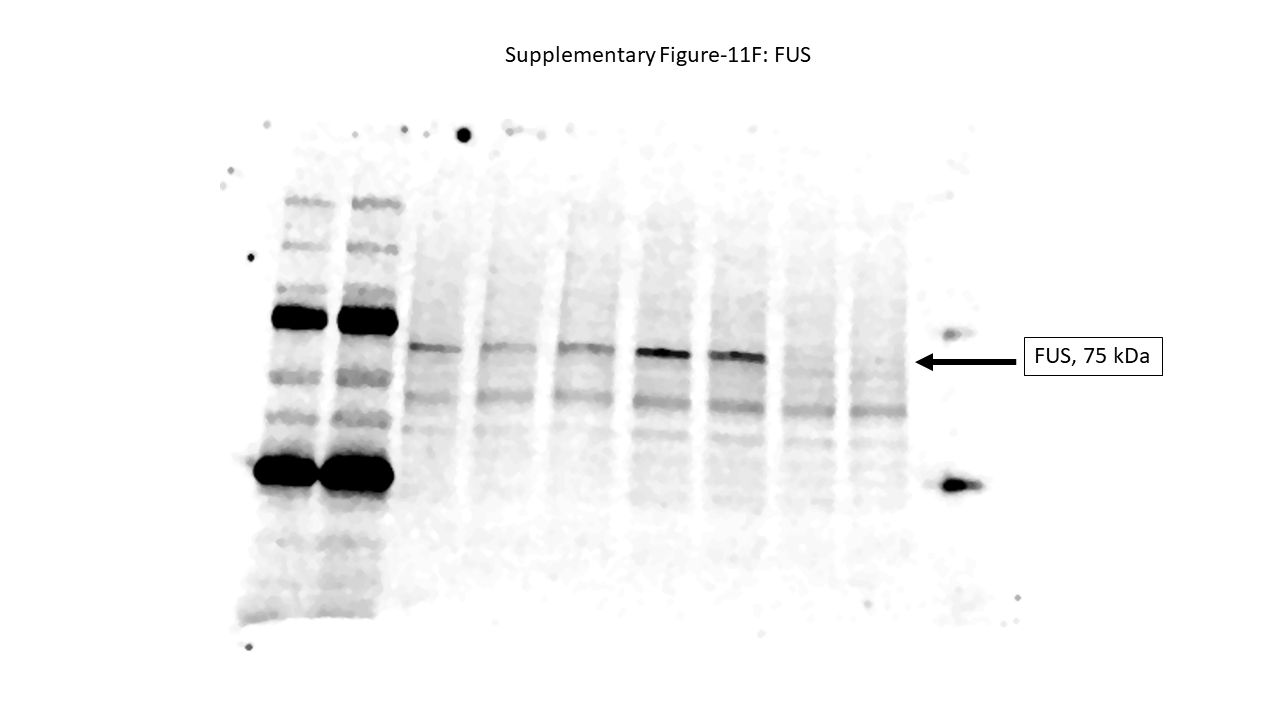

Supplement: Supplementary file 4 — Source Data [file 41467_2019_11777_MOESM4_ESM.zip › Source data/Slide14.TIF]

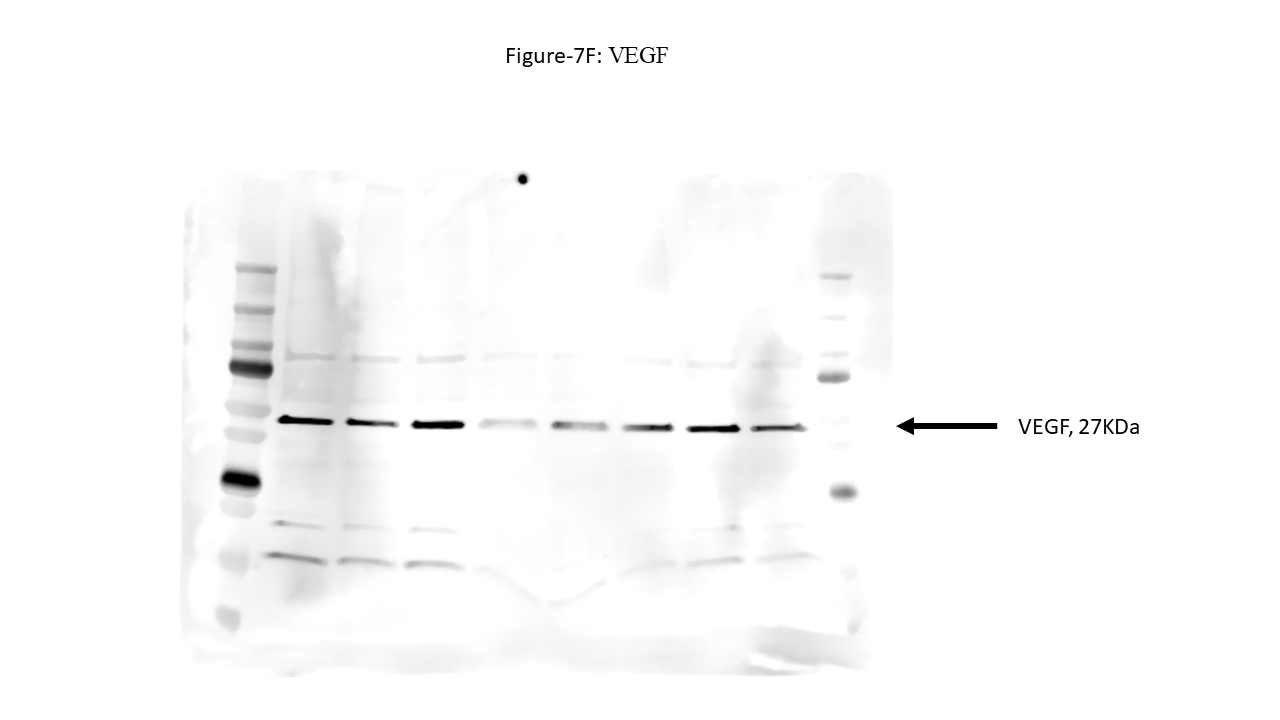

Supplement: Supplementary file 4 — Source Data [file 41467_2019_11777_MOESM4_ESM.zip › Source data/Slide6.TIF]

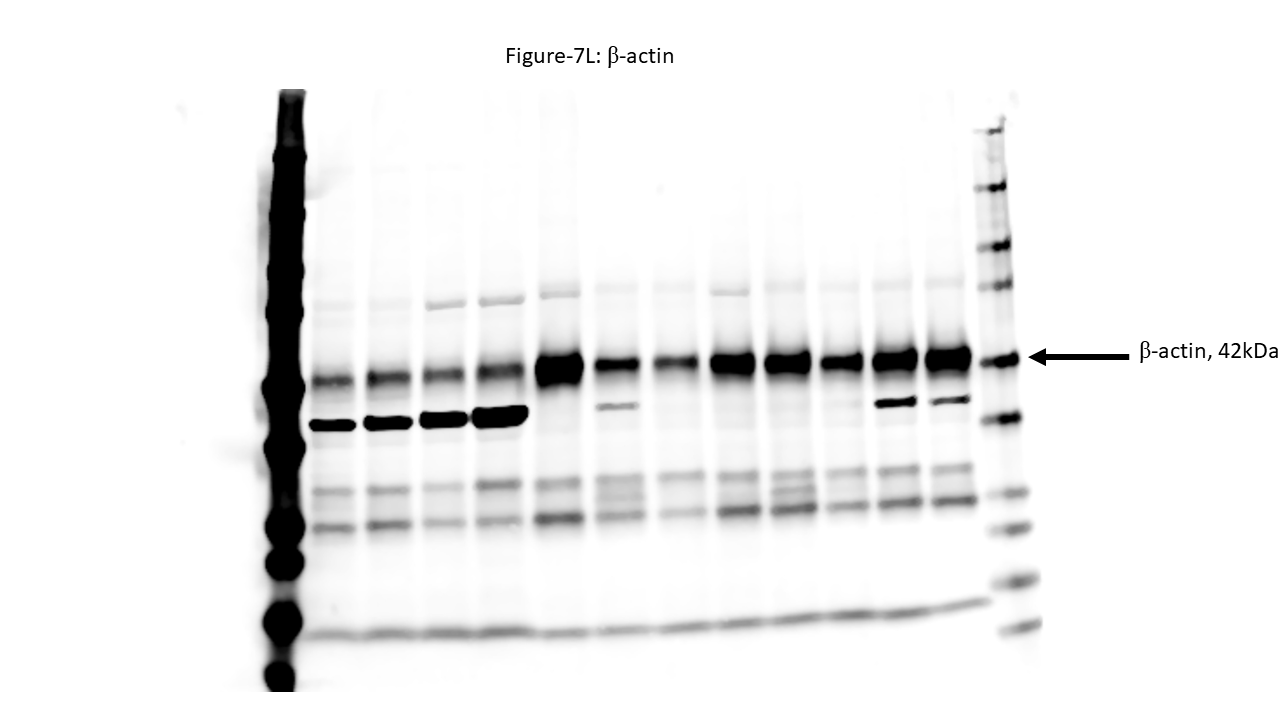

Supplement: Supplementary file 4 — Source Data [file 41467_2019_11777_MOESM4_ESM.zip › Source data/Slide5.TIF]

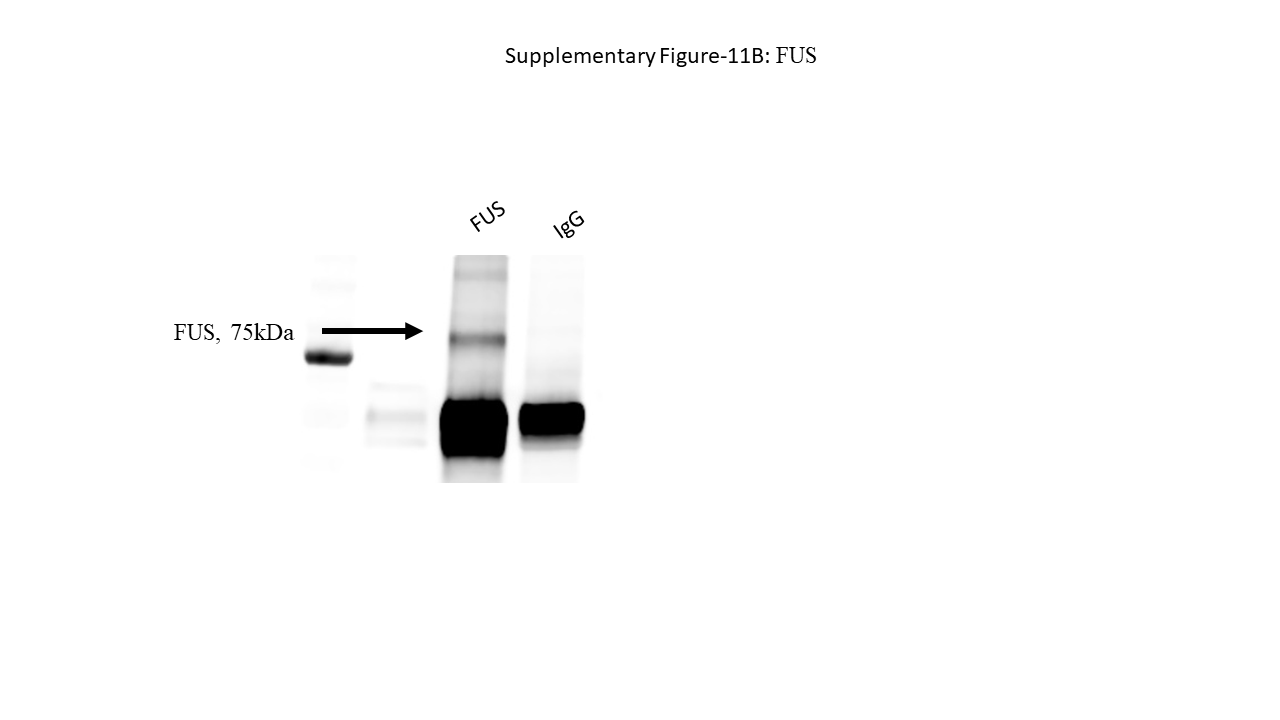

Supplement: Supplementary file 4 — Source Data [file 41467_2019_11777_MOESM4_ESM.zip › Source data/Slide12.TIF]

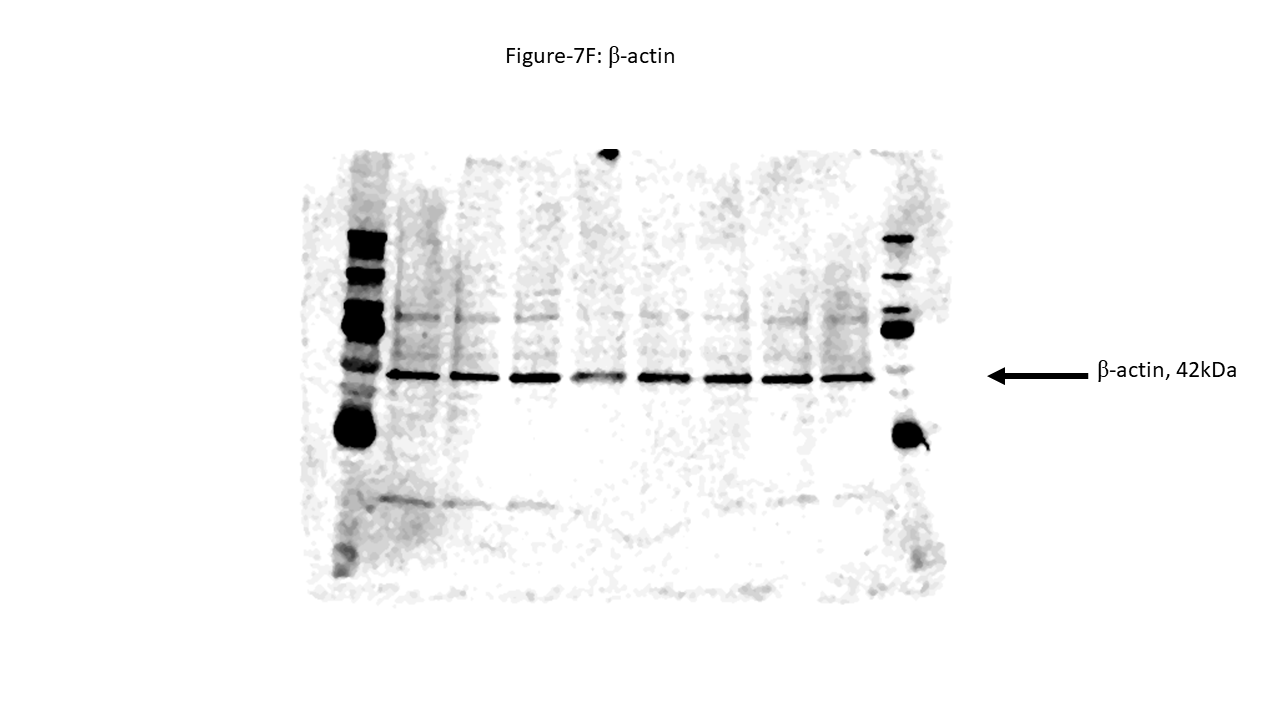

Supplement: Supplementary file 4 — Source Data [file 41467_2019_11777_MOESM4_ESM.zip › Source data/Slide7.TIF]

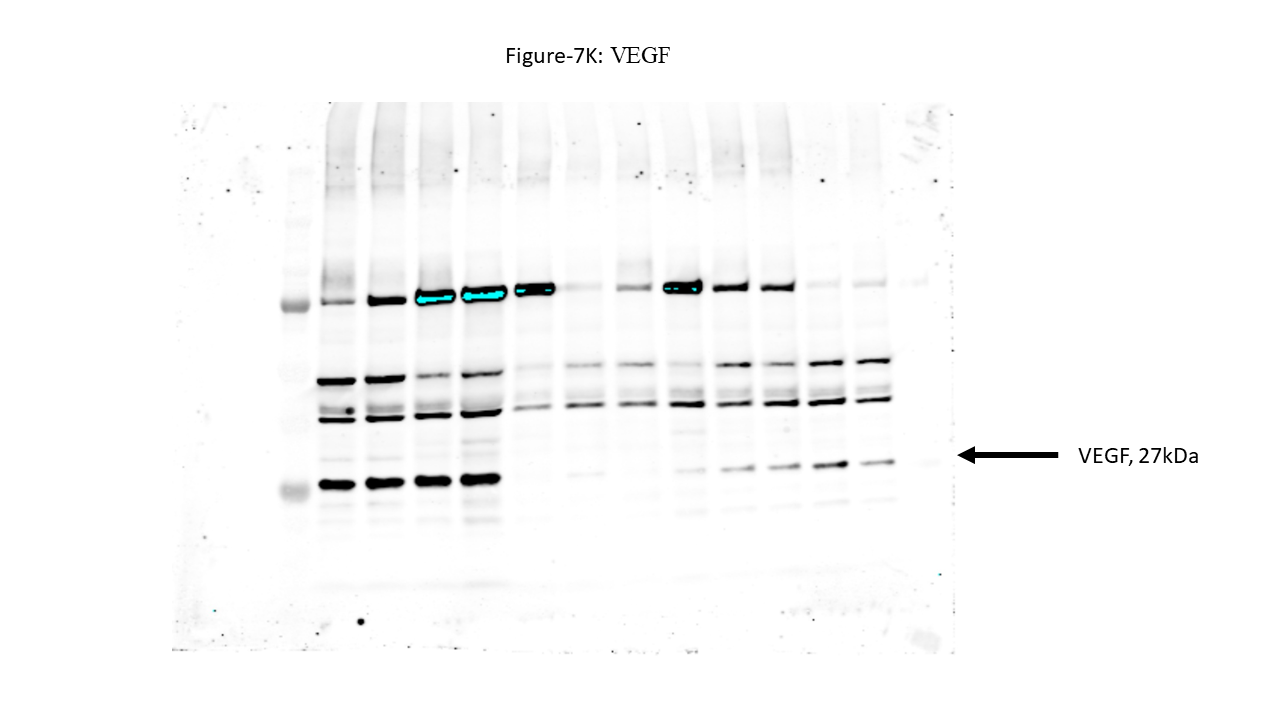

Supplement: Supplementary file 4 — Source Data [file 41467_2019_11777_MOESM4_ESM.zip › Source data/Slide4.TIF]

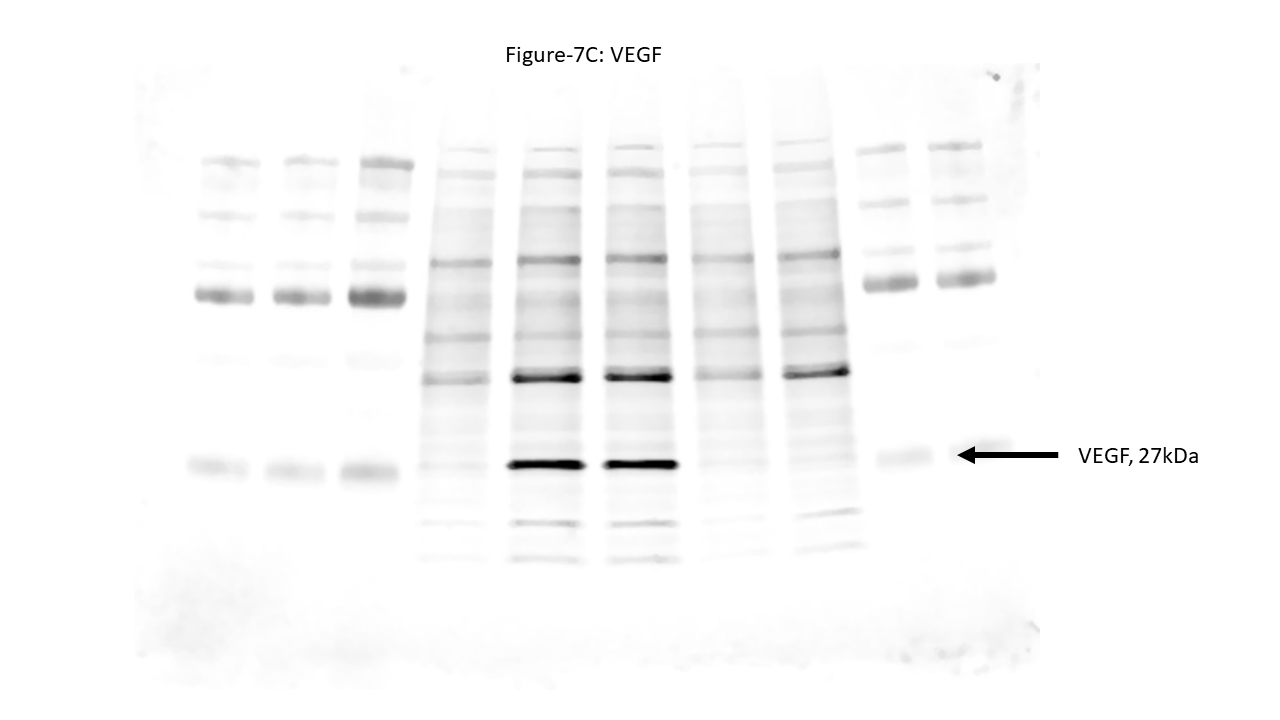

Supplement: Supplementary file 4 — Source Data [file 41467_2019_11777_MOESM4_ESM.zip › Source data/Slide2.TIF]

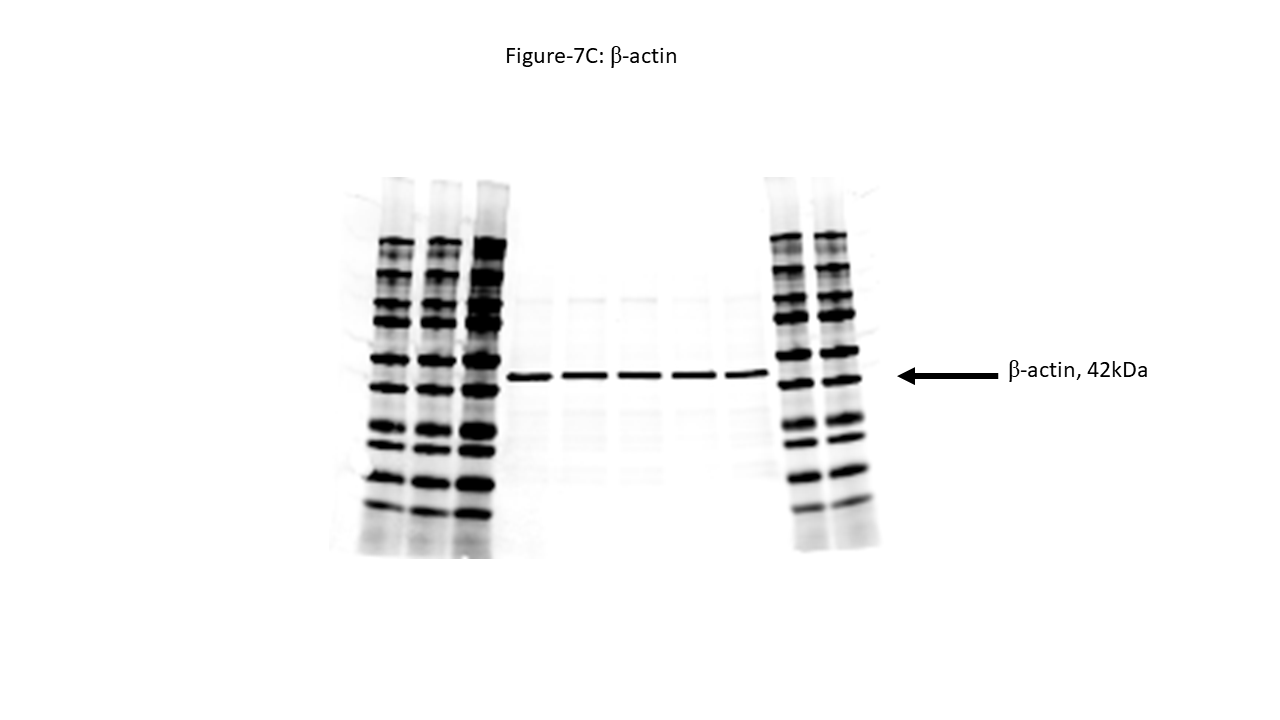

Supplement: Supplementary file 4 — Source Data [file 41467_2019_11777_MOESM4_ESM.zip › Source data/Slide3.TIF]

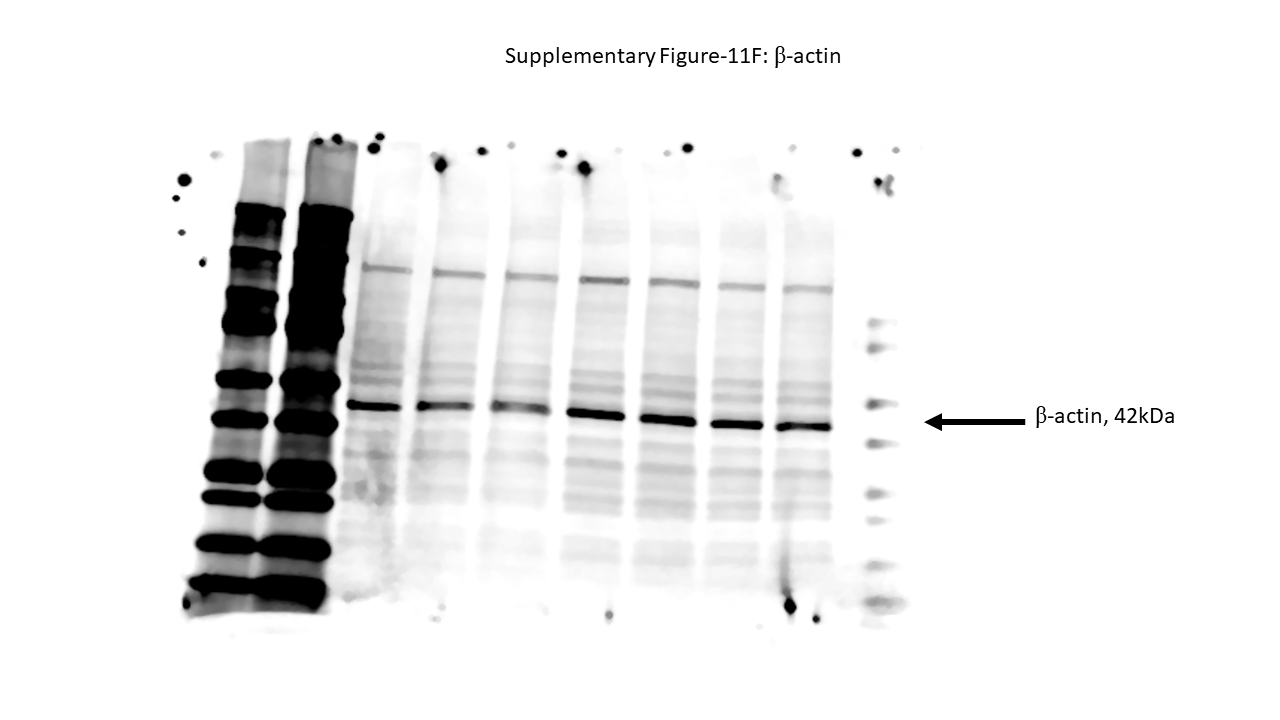

Supplement: Supplementary file 4 — Source Data [file 41467_2019_11777_MOESM4_ESM.zip › Source data/Slide15.TIF]

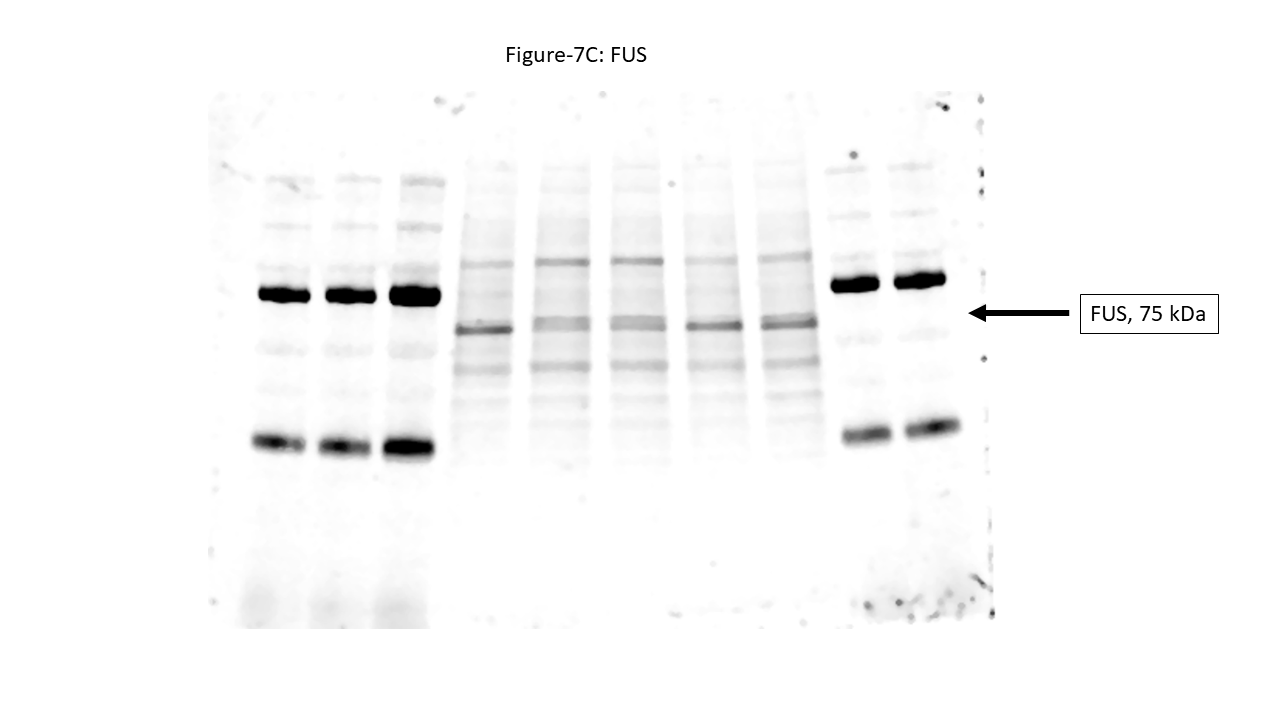

Supplement: Supplementary file 4 — Source Data [file 41467_2019_11777_MOESM4_ESM.zip › Source data/Slide1.TIF]
